# Magnesium-doped bioactive glass enhances bone regeneration by reversing replicative senescence of human dental pulp stem cells in bone defect therapy

**DOI:** 10.1093/rb/rbaf105

**Published:** 2025-10-25

**Authors:** Xin Yan, Xiangdong Li, Qi Zhang, Xinlin He, Qi Chen, Sui Mai

**Affiliations:** Hospital of Stomatology, Sun Yat-sen University, Guangzhou 510055, China; Guanghua School of Stomatology, Sun Yat-sen University, Guangzhou 510055, China; Guangdong Provincial Key Laboratory of Stomatology, Institute of Stomatology, Sun Yat-sen University, Guangzhou 510080, China; Hospital of Stomatology, Sun Yat-sen University, Guangzhou 510055, China; Guanghua School of Stomatology, Sun Yat-sen University, Guangzhou 510055, China; Guangdong Provincial Key Laboratory of Stomatology, Institute of Stomatology, Sun Yat-sen University, Guangzhou 510080, China; Hospital of Stomatology, Sun Yat-sen University, Guangzhou 510055, China; Guanghua School of Stomatology, Sun Yat-sen University, Guangzhou 510055, China; Guangdong Provincial Key Laboratory of Stomatology, Institute of Stomatology, Sun Yat-sen University, Guangzhou 510080, China; Hospital of Stomatology, Sun Yat-sen University, Guangzhou 510055, China; Guanghua School of Stomatology, Sun Yat-sen University, Guangzhou 510055, China; Guangdong Provincial Key Laboratory of Stomatology, Institute of Stomatology, Sun Yat-sen University, Guangzhou 510080, China; Hospital of Stomatology, Sun Yat-sen University, Guangzhou 510055, China; Guanghua School of Stomatology, Sun Yat-sen University, Guangzhou 510055, China; Guangdong Provincial Key Laboratory of Stomatology, Institute of Stomatology, Sun Yat-sen University, Guangzhou 510080, China; Hospital of Stomatology, Sun Yat-sen University, Guangzhou 510055, China; Guanghua School of Stomatology, Sun Yat-sen University, Guangzhou 510055, China; Guangdong Provincial Key Laboratory of Stomatology, Institute of Stomatology, Sun Yat-sen University, Guangzhou 510080, China

**Keywords:** replicative senescence, human dental pulp stem cells, magnesium-doped bioactive glass, osteogenic differentiation, IKBKGP1, NF-κB

## Abstract

Human dental pulp stem cells (hDPSCs) exhibit replicative senescence during *in vitro* expansion, leading to a reduction in osteogenic differentiation capacity and thereby limiting their potential for bone defect regeneration. Magnesium ion (Mg^2+^), one of the most abundant divalent cations in the human body, is involved in numerous physiological processes. Mg^2+^ deficiency has been closely associated with bone fragility and various systemic aging-related diseases, underscoring its critical role in aging and bone metabolism. However, the effects of Mg^2+^ on mesenchymal stem cells (MSCs) replicative senescence remain poorly understood. In this study, we developed magnesium-doped bioactive glass (Mg-BG) powder with a graded magnesium doping ratio through the sol-gel method, and characterized the pore structure and ion release profiles of each Mg-BG group. We demonstrated that 20 Mg-BG (Mg-BG containing 20 mol% MgO) can effectively reverse the replicative senescence of hDPSCs, improve mitochondrial function, reduce ROS levels and enhance the expression of surface markers associated with differentiation, migration and adhesion in replicatively senescent hDPSCs, thereby enhancing their osteogenic differentiation potential. Furthermore, *in vivo* experiments using a rat calvarial defect model also confirmed that 20 Mg-BG significantly enhances bone defect repair mediated by replicatively senescent hDPSCs. Mechanistically, we found that the IKBKGP1-mediated NF-κB pathway may play a key role in this process, as revealed by transcriptome sequencing. These findings indicate that Mg-BG could serve as an effective, innovative approach to reverse replicative senescence in hDPSCs and enhance their bone defect repair capabilities.

## Graphical Abstract

**Figure:**
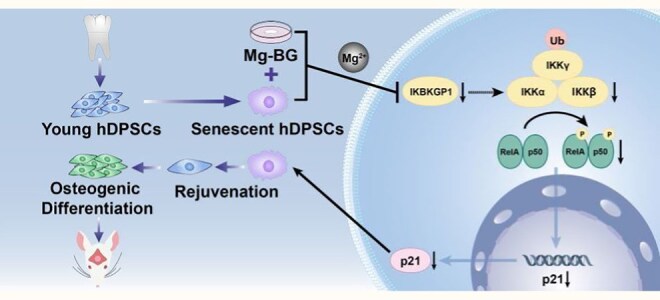

## Introduction

Jaw defects resulting from oral trauma, infection, tumors and other causes pose significant clinical challenges, severely impacting patients’ aesthetics, speech and masticatory function [1]. Over two million patients worldwide undergo bone defect transplantation annually, including procedures for jaw reconstruction [2]. Currently, guided bone regeneration and autologous and allogeneic bone grafting are the primary approaches for enhancing bone volume; however, these methods are associated with substantial drawbacks, including prolonged healing time, extensive surgical trauma and multiple donor site complications [3, 4]. These drawbacks underscore the urgent need for alternative strategies capable of achieving predictable and functional bone regeneration. Bone tissue engineering, facilitated by mesenchymal stem cells (MSCs) with robust osteogenic differentiation potential, holds significant promise for advancements in bone regeneration technologies [5, 6].

To achieve optimal bone regeneration, among various stem cell sources, human dental pulp stem cells (hDPSCs) are highly valued due to their ease of collection and strong regenerative potential for bone, cartilage and other tissues [7]. Their contribution to bone repair primarily relies on osteogenic differentiation, through which hDPSCs differentiate into osteoblast-like cells capable of depositing mineralized matrix and promoting new bone formation within defect sites [8]. Studies have demonstrated that hDPSCs can effectively repair periodontal bone defects in a miniature pig periodontitis model associated with jaw defects [9]. Maintaining a high osteogenic differentiation capacity in hDPSCs is critical for successful bone tissue formation and effective bone regeneration [10, 11]. However, bone defect repair requires a substantial number of seed cells [12], and hDPSCs are relatively scarce within the dental pulp, necessitating *in vitro* expansion to attain the required cell quantity [13]. MSCs, including hDPSCs, undergo significant replicative senescence during *in vitro* expansion and proliferation [13]. MSCs replicative senescence is characterized by an irreversible cell cycle arrest in cells that were previously capable of replication [14], leading to profound changes in stem cell phenotype [15, 16], reduced proliferative capacity [17], impaired multipotency—particularly a decline in osteogenic differentiation, and diminished immunoregulatory function [18, 19]. The loss of differentiation potential following *in vitro* expansion has long been a major bottleneck, hindering large-scale bone defect repair and severely restricting the clinical application of MSCs-based therapies [20].

Magnesium ions (Mg^2+^), one of the most prevalent divalent cations in the human body, play a key role in maintaining intracellular homeostasis and the proper functioning of organs [21]. Intracellular Mg^2+^ concentrations decrease in an age-dependent manner [22]. Studies have indicated that Mg^2+^ deficiency can lead to age-related characteristics, including mitochondrial dysfunction [23], the production of pro-inflammatory molecules such as interleukin-1 (IL-1) [24], interleukin-6 (IL-6) [25] and tumor necrosis factor-α (TNF-α) [26] within the senescence-associated secretory phenotype (SASP), increased generation of reactive oxygen species (ROS) [27, 28], reduced cell proliferative capacity and elevated expression of biomarkers associated with aging and telomere attrition [29]. Based on this evidence, we hypothesize that Mg^2+^ is intricately associated with cellular senescence, yet the impact of Mg^2+^ on MSCs senescence remains poorly understood.

To introduce Mg^2+^ into the microenvironment of hDPSCs and investigate whether it can alleviate replicative senescence and promote osteogenic differentiation, we sought a carrier bioactive material capable of effectively releasing Mg^2+^ in the hDPSCs’ culture environment. Bioactive glass (BG), an inorganic bone biomaterial with excellent bioactivity and biocompatibility, was employed to carry and release Mg^2+^ [30]. Meta-analysis results indicate that BG-based implant materials exhibit superior regenerative potential for periodontal bone tissue compared to enamel matrix protein derivatives and demineralized freeze-dried allogeneic bone grafts [31]. As a mesoporous material, BG synthesized via second-generation sol-gel technology features distinct pores formed by sol accumulation, a larger specific surface area and a faster apatite deposition rate compared to 45S5 Bioglass^®^ [32]. This unique structure enables BG to load and slowly release soluble ions and molecules, including critical concentrations of calcium, phosphorus, silicon and other ions, at a rate that supports cell proliferation and differentiation [33]. Therefore, we speculate whether magnesium-doped bioactive glass can release an appropriate amount of magnesium ions to reverse the replicative senescence of hDPSCs.

Currently, the osteogenic potential of magnesium-containing bioactive materials shows some limitations in terms of the efficiency and consistency of bone formation [34], which may be attributed to the Mg^2+^ release behavior regulated by these materials: low-dose Mg^2+^ (<10 mM) release can enhance bone formation, but further optimization is needed to fully maximize their regenerative capacity [35], whereas local high concentrations of Mg^2+^ may lead to the formation of degradation products, such as magnesium hydroxide, which interfere with calcium-mediated bone repair and regeneration processes [36], thereby hindering tissue healing. However, the function and optimal magnesium doping ratio of Mg-BG in promoting the osteogenic differentiation of hDPSCs, as well as the underlying mechanisms, remain unclear. In our study, we propose that Mg-BG can enhance the osteogenic differentiation ability, and the osteogenic capacity of Mg-BG is related to the Mg doping ratio. More importantly, we suggest that the bone regeneration efficacy of Mg-BG combined with hDPSCs is driven by its ability to reverse hDPSCs’ replicative cellular senescence. Additionally, we identified the most suitable Mg ratio that reverses replicative senescence in hDPSCs and subsequently enhances their osteogenic potential, while also explored the molecular mechanisms through which Mg-BG mitigates replicative senescence.

Therefore, this study proposes the hypothesis that doping an optimal amount of Mg in BG can reverse the replicative senescence of hDPSCs, thereby enhancing their osteogenic differentiation capacity. In this study, Mg-BG was synthesized using a modified sol-gel method, and Mg-BG with a gradient molar ratio was developed. A significant impairment in the osteogenic differentiation capacity of replicatively senescent hDPSCs was confirmed. Through both *in vitro* and *in vivo* experiments, we investigated the effect of Mg-BG on the replicative senescence of hDPSCs, and its effect on the osteogenic differentiation capacity of replicatively senescent hDPSCs. After confirming these findings, we further explored the molecular mechanisms through which Mg-BG reverses hDPSCs’ senescence and explored the crucial role of the IKBKGP1-mediated NF-κB pathway in this process.

## Materials and methods

### Synthesis of Mg-BG powders

Mg-BG was prepared via the sol-gel method [37]. Tetraethyl orthosilicate (TEOS; Aladdin Co., Ltd, China), triethyl phosphate (TEP; Aladdin Co., Ltd, China), calcium nitrate (CN; Aladdin Co., Ltd, China) and magnesium nitrate (MN; Aladdin Co., Ltd, China) functioned as precursors for SiO_2_, P_2_O_5_, CaO and MgO. Hydrochloric acid was initially dissolved in deionized water with agitation to act as a catalyst. Subsequently, TEOS, TEP, CN and MN were incorporated into sequence to the acidic solution, with 30-min intervals between each addition as indicated in Table 1. The sol was stirred at room temperature for 3 h, followed by aging at 37°C and 60°C and drying at 120°C. The xerogel was then sintered at 600°C, and the resulting Mg-BG powder was ball-milled and sieved for further experiments.

**Table 1:** Composition of Mg-BG powders (mol%).

Sample name	MgO	CaO	SiO_2_	P_2_O_5_
0 Mg-BG	0	36	60	4
6 Mg-BG	6	30	60	4
15 Mg-BG	15	21	60	4
20 Mg-BG	20	16	60	4
36 Mg-BG	36	0	60	4

### Characterization of Mg-BG powders

The five Mg-BG cement pastes with varying component ratios were analyzed by X-ray diffraction (XRD; Empyrean, NLD) and Fourier-transform infrared spectroscopy (FTIR; Nicolet Co., USA) to examine their chemical functionalities. Surface and cross-sectional morphologies were observed using field emission scanning electron microscopy (FE-SEM; Merlin Carl Zeiss Jena, GER), while micromorphologies and crystallographic arrangements were evaluated via transmission electron microscopy (TEM; Talos F200X, USA).

Nitrogen adsorption-desorption isotherms were obtained at 77 K using a surface area and porosity analyzer (Kubo-X1000, bjbuilder, CHN). The pore size distribution and specific surface area of Mg-BG were evaluated by the Barrett–Joyner–Halenda (BJH) and Langmuir methods.

Mg-BG powder extracts were prepared according to ISO 10993-12: 2021, and the supernatant was mixed with standard culture medium at a 1:32 ratio (based on previous reports) [37]. Mg^2+^ concentration was measured by inductively coupled plasma emission spectrometry (ICP-OES 725ES, Agilent), Ca^2+^ concentration by a calcium ion meter (PXSJ-216F, INESA Scientific Instrument Co., Ltd, CHN) and pH by a pH meter (S220, METTLER TOLEDO, USA).

### Cell culture of young and replicative senescent hDPSCs

Dental pulp was collected from third molars or orthodontically extracted premolars of three donors (*n* = 3; aged 16–22 years) under the approval of the Ethics Committee of the Hospital of Stomatology, Sun Yat-sen University (GHKQ-202502-K04-01). The tissue was digested with 3 mg/ml type I collagenase and 4 mg/mL dispase (both from Sigma-Aldrich, St Louis, MO, USA) at 37°C for 20–40 min. The cell suspension was plated in 25 cm^2^ flasks and cultured in α-modified minimum essential medium (α-MEM; GIBCO, USA) with 10% fetal bovine serum (FBS; GIBCO, Grand Island, NY, USA), 100 U/mL penicillin and 100 µg/ml streptomycin (PS) at 37°C in 5% CO_2_. Cells at passage 4 (P4) were classified as young hDPSCs, while passage 10 (P10) cells were used as replicatively senescent hDPSCs.

### Identification of hDPSCs and replicative cellular senescence of hDPSCs

hDPSCs were identified by cell adhesion, growth morphology, immunophenotypic profile and osteogenic differentiation potential. Cell morphology was observed with an inverted microscope (Axio Zeiss, GER). The immunophenotypic profile was evaluated by flow cytometry. hDPSCs (P4) were collected with 0.25% trypsin-EDTA (Gibco, USA), washed twice with phosphate-buffered saline (PBS) and incubated with fluorophore-conjugated antibodies targeting surface markers: positive (CD90, CD105, CD106, CD146) and negative (CD45, CD14) markers (Biolegend, USA; Abclonal, CHN). After 30 min of incubation at 4°C in the dark, cells were washed with PBS. Flow cytometry was conducted using the BD LSRFortessa system (BD Biosciences, USA), and data were analyzed with FlowJo (version 10.5.3). Osteogenic differentiation was assessed by alizarin red staining (ARS) (as shown below).

The hDPSCs were subcultured in standard culture medium to passages 4, 6, 8 and 10. Replicative senescence was identified by observing cell adhesion and growth morphology, SA-β-gal staining and qRT-PCR analysis of senescence-related genes. Osteogenic differentiation ability in replicatively senescent hDPSCs was assessed by alkaline phosphatase (ALP) staining, ARS staining and qRT-PCR analysis of osteogenic differentiation-related genes (as shown below).

### Cell proliferation assay

hDPSCs were seeded in 96-well plates at 2 × 10³ cells per well and treated with Mg-BG extracts mixed with standard culture medium at a 1:32 ratio. The control group received PBS mixed with complete medium at the same ratio. After 1, 3, 5 and 7 days, a CCK-8 assay (Dojindo, CHN) was performed, and optical density was measured at 450 nm.

### Osteogenic differentiation induction culture

To induce osteogenic differentiation, young and replicatively senescent hDPSCs were treated with Mg-BG extracts, MTA (Dentsply, GER) as a positive control or PBS as a negative control, mixed with osteogenic differentiation medium at a 1:32 ratio.

The osteogenic induction medium composition is as follows: α-MEM supplemented with 10% FBS, 1% PS, 10 mM β-glycerophosphate (β-GP), 100 nM dexamethasone (Dex), 1.8 mM potassium dihydrogen phosphate (KH_2_PO_4_) and 50 μM ascorbic acid (AA). The medium was replaced every 2 days, and the cells were cultured under standard conditions (37°C, 5% CO_2_) for the designated experimental period to promote osteogenic differentiation.

### SA-β-gal staining

Young and replicatively senescent hDPSCs were cultured with Mg-BG extracts, MTA (positive control) or PBS (negative control) mixed with standard culture medium at a 1:32 ratio. After 7 days, SA-β-Gal staining was performed using the SA-β-Gal staining kit (Beyotime, CHN). Cells were fixed with 4% formaldehyde at room temperature for 30 min, stained with X-gal solution for 24 h at 37°C (no CO_2_) and observed under a color-inverted fluorescence microscope (Axio Zeiss, GER). The ratio of positively stained cells was calculated using ImageJ.

### qRT-PCR analysis

To assess replicative cellular senescence of hDPSCs, RNA was extracted after three days of culture in standard medium. To explore the impact of Mg-BG on cellular senescence and the role of the NF-κB pathway, cell treatment and collection were performed as described in section qRT-PCR analysis, with RNA extraction after 7 days of culture. Additionally, to evaluate the effect of Mg-BG on osteogenic differentiation, treatment and collection followed the procedure in section SA-β-gal staining, with RNA extraction after 7 days.

RNA from each group of hDPSCs was isolated using an RNA extraction kit (ES Science, CHN) and reverse-transcribed into complementary DNA (cDNA) using the RT reagent kit (Takara, JPN). The cDNA served as a template for mRNA expression analysis using a qRT-PCR system (QuantStudio™ 7 Flex, Applied Biosystems, USA). In brief, equal amounts of cDNA from each group were combined with SYBR Green (Vazyme, CHN), and forward and reverse primers for qRT-PCR analysis. Primer sequences are provided in Supplementary Table S1.

### Western blot analysis

To investigate the impact of Mg-BG on hDPSCs’ cellular senescence and the role of the NF-κB pathway in this process, cell treatment and collection were performed as described in section qRT-PCR analysis, with protein extracted after 7 days of culture. Additionally, to evaluate the effect of Mg-BG on osteogenic differentiation, the same procedures were followed, with protein extracted after 7 days of culture.

Protein from each group of hDPSCs was extracted using radio immunoprecipitation assay (RIPA) lysis buffer (Beyotime, CHN), containing 1 mM phenylmethanesulfonyl fluoride (PMSF) (CWBIO, CHN) and 1 mM Phosphatase Inhibitor Cocktail (CWBIO, CHN). The protein concentration was determined using the BCA Protein Assay kit (CWBIO, CHN). Proteins were separated by SDS-PAGE (ACE, CHN) and transferred to a PVDF membrane (0.22 μm, Merck Millipore, USA), blocked with 5% skim milk (Biofrox, CHN) and incubated overnight at 4°C with primary antibodies: anti-p16, anti-p21, anti-p53 (CST, USA), anti-DSPP, anti-RUNX2, anti-COL1 (Santa Cruz, USA; Proteintech, USA). The membrane was then incubated with an HRP-conjugated secondary antibody at room temperature for 1 h. Protein bands were visualized using the enhanced chemiluminescence (ECL) substrate kit (Merck Millipore, USA) and a chemiluminescence imaging system (Bio-Rad, USA). Protein expression was quantified with ImageJ and normalized to the gray value of GAPDH.

### Intracellular ROS measurement

To investigate the impact of Mg-BG on senescent hDPSCs intracellular ROS levels, the cell treatment and collection procedures were conducted as described in section qRT-PCR analysis, with ROS levels detected after 7 days of culture. Intracellular ROS levels were assessed using a ROS kit (Beyotime, CHN). The fluorescence of dichlorofluorescein was measured by flow cytometry. Flow cytometry assessment was conducted using the BD LSRFortessa system.

### Mitochondria membrane potential

To investigate the impact of Mg-BG on mitochondrial function in senescent hDPSCs, cell treatment and collection were performed as described in section qRT-PCR analysis, with mitochondria membrane potential (MMP) levels detected after 7 days of culture. Mitochondrial MMP was assessed using a JC-1 assay kit (Beyotime). In the first approach, cells were washed twice with PBS and incubated with the JC-1 staining solution at 37°C for 20 min, then, washed twice with JC-1 dilution buffer and examined with a confocal microscope (LSM980, Zeiss, GER). In the second approach, cells were digested with Tryple (Gibco, USA) for 2 min, and the reaction was stopped by adding serum-containing medium. After centrifugation at 1000 rpm for 5 min, cells were incubated with JC-1 staining solution at 37°C for 20 min and washed twice with JC-1 dilution buffer. Flow cytometry was conducted using the BD LSRFortessa system.

### Cell surface marker detection

To investigate the impact of Mg-BG on adhesion, migration and differentiation-related cell surface markers in senescent hDPSCs, cell treatment and collection were performed as described in section qRT-PCR analysis, with cell surface marker expression detected after 7 days of culture. Cells were digested with Tryple (Gibco, USA) for 2 min, and the reaction was terminated by adding serum-containing medium twice. After centrifugation at 1000 rpm for 5 min, cells were incubated with CD90 (Biolegend, USA), CD106 (Abclonal, USA) and CD146 (Biolegend, USA) at 37°C for 20 min, followed by two washes with staining buffer (Biolegend, USA). Flow cytometry was conducted using the BD LSRFortessa system.

### ALP and ARS staining

To investigate the impact of Mg-BG on the osteogenic differentiation potential of hDPSCs, cell culture and collection were performed as described in section qRT-PCR analysis. ALP staining was conducted after 7 days of culture. Cells were washed twice with PBS, fixed for 30 min and stained for 30 min according to the ALP staining kit protocol (PH Biotechnology, China). ARS staining was performed after 14 or 21 days of culture. Cells were fixed with 4% paraformaldehyde (PFA), rinsed twice with ddH_2_O and stained with 1% alizarin red dye (Beyotime, CHN). Mineralized nodules were observed under an optical microscope, and the mean intensity density value of each well was calculated using ImageJ.

### Creation of cranial defects in rats

The animal procedures were approved by the Sun Yat-Sen University Laboratory Animal Center (2024000041), and all procedures were conducted following the guidelines of the Sun Yat-Sen University Animal Care and Use Committee. Male Sprague-Dawley rats (6 weeks, 250–280 g) were used, with the rats divided equally into six groups: Blank, hDPSCs (P4) + HA-TCP, hDPSCs (P10) + HA-TCP, hDPSCs (P10) + MTA, hDPSCs (P10) + 0 Mg-BG and hDPSCs (P10) + 20 Mg-BG. A midline incision was made along the scalp, and the subcutaneous tissue was dissected to expose the skull. A 5 mm full-thickness skull defect was created using a trephine drill, and the dura mater was irrigated with saline to prevent damage. Except for the blank control group, material cement powders from each group were placed in the defect area, and 1 × 10^5^ cells from each group were seeded. The surgical incisions were closed with 3–0 sutures. After 4 weeks of healing, the skulls, hearts, livers, spleens, lungs and kidneys were collected, fixed in 4% PFA and analyzed using micro-computed tomography (CT), histological and immunological techniques.

### Microcomputed tomography analysis

The development of new osseous tissue was assessed using a micro-CT scanner (SCANCO, CH) with an X-ray voltage of 70 kV, current of 200 μA and a scanning resolution of 10 μm. Three-dimensional reconstruction of the skull samples was performed using RadiAnt DICOM Viewer software. The micro-CT software was used to generate three-dimensional reconstructions, and the following parameters of the region of interest (ROI) were measured: bone volume fraction (BV/TV), trabecular number (Tb.N), trabecular thickness (Tb.Th), trabecular separation (Tb.Sp) and connectivity density (Conn.D).

### H&E staining

The hearts, livers, spleens, lungs and kidneys harvested from rats in the six groups were fixed with 4% PFA for 24 h, embedded in paraffin and sliced into 4 μm thick sections. The skulls from the six groups were fixed in 4% PFA for 24 h, decalcified for 3 weeks, embedded in paraffin, and sliced into 4 μm thick specimens. H&E staining was then performed using an H&E staining kit (Biosharp, CHN).

### Immunohistochemistry

The rat skulls were fixed, decalcified, embedded and sliced following the procedure described in section immunohistochemistry. To determine the distribution of Col1, RUNX2 and OCN expression in bone tissue, the slides were blocked with 3% bovine serum albumin (BSA) and incubated overnight at 4°C with anti-Col1 (Proteintech, USA), anti-RUNX2 (Proteintech, USA) and anti-OCN (Proteintech, USA) antibodies. After washing, the slides were incubated with a secondary antibody for 30 min. The immune response was visualized using a DAB kit (Gene Tech, CHN), and the sections were stained with methylgreen. Observations were made under a color-inverted fluorescence microscope (Axio Zeiss, GER).

### RNA-seq

RNA-seq consisted of three groups: Passage 4 young hDPSCs cultured in PBS mixed with the standard culture medium at a 1:32 ratio (P4 group), Passage 10 replicative senescent hDPSCs cultured in PBS mixed with the standard culture medium at a 1:32 ratio (P10 group), and Passage 10 replicative senescent hDPSCs cultured in 20 Mg-BG extracts mixed with the standard culture medium at a 1:32 ratio (20 Mg-BG group). A total of 1 × 10^6^ cells were plated in 10 cm dishes, collected after 7 days of culture and RNA-seq was performed to investigate the transcriptome profiles of hDPSCs in each group. RNA-seq libraries were constructed using the Illumina TruSeq RNA Library Prep Kit, including poly(A) enrichment, RNA fragmentation, cDNA synthesis, adapter ligation, PCR amplification and library construction. Sequencing was performed on the Illumina NovaSeq X Plus platform, generating 150 bp paired-end reads. The accession number for the RNA-seq data in this study is PRJNA1218403.

### Statistical analysis

All experiments were performed in triplicate, with data presented as the mean ± standard deviation. Statistical analysis was conducted using a one-way analysis of variance (ANOVA) for comparisons among three groups and a Student’s *t*-test for comparisons between two groups, using GraphPad Prism 8.0 (GraphPad Software). Statistical significance was defined as *P < 0.05, **P < 0.01 and ***P < 0.001.

## Results

### Characterization of Mg-BG powders

**Figure 1:**
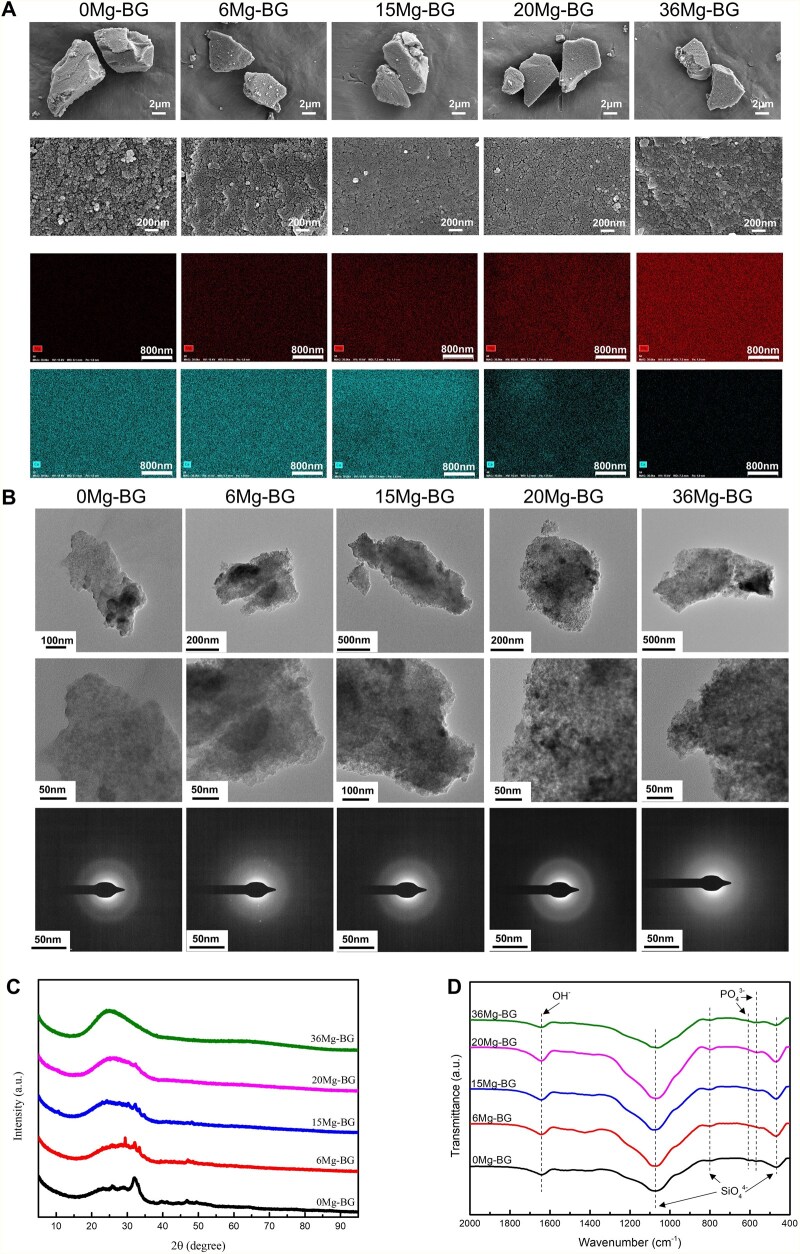
Physical and chemical characteristics of the Mg-BG powders. (**A**) SEM morphology and elemental mapping of Mg-BG; (**B**) TEM internal structure of Mg-BG; (**C**) X-ray diffraction patterns for phase identification of Mg-BG; (**D**) FTIR spectra showing the molecular structure of Mg-BG.

Micro-nano Mg-BG powders were prepared through the sol-gel method. The surface morphologies and microstructures of each group were observed using SEM and TEM, respectively. All samples (0 Mg-BG, 6 Mg-BG, 15 Mg-BG, 20 Mg-BG and 36 Mg-BG) exhibited irregular, polygonal micron-sized particles formed from nanoscale components, characterized by a porous structure and rough surface (Figure 1A and B). Notably, diffraction patterns indicated that 0 Mg-BG exhibited a slight tendency to crystallize, whereas all other groups, except for 0 Mg-BG, presented amorphous diffraction rings, which corresponded to wide halos and dispersed rings (Figure 1B). Aligned with the diffraction patterns, the XRD spectra demonstrated that 0 Mg-BG exhibited a slight tendency towards crystallization, as indicated by the diffraction peak around 33°. In contrast, all other Mg-BG groups showed no distinct crystalline features and displayed a “mantou peak” near 2θ = 28°, corresponding to the primary peak of silicon dioxide crystals, suggesting an amorphous state (Figure 1C). FTIR spectra demonstrated that all Mg-BG powder groups exhibited similar structures, featuring prominent bands at 475, 800 and 1090 cm^−1^, corresponding to the flexural and tensile modes of the Si-O-Si bond in SiO_2_. The double bands at 562 and 603 cm^−1^ were attributed to the P-O bonds of P_2_O_5_, while the band at 1640 cm^−1^ was linked to the O-H stretching modes of H_2_O molecules (Figure 1D).

The above results indicate that the incorporation of magnesium promotes the formation of an amorphous, noncrystalline structure in BG, which indicated that magnesium incorporation can reduce the crystallization tendency of Mg-BG powder. Furthermore, as the magnesium content increases, the proportion of MgO in the Mg-BG powder rises, while the CaO content decreases. Overall, the addition of magnesium exerts minimal influence on the chemical composition and microstructure of BG [37].

Notably, the inorganic composition and Mg/Ca doping ratio of the Mg-BG system partially mimic the characteristics of natural bone. Bone mineral is primarily composed of hydroxyapatite (HA), with silicate and phosphate components that are chemically similar to BG [38, 39]. Magnesium, a trace element comprising approximately 0.5–1 wt% of bone’s inorganic content, plays a key role in regulating crystal structure and mechanical properties [40]. However, the distribution of magnesium in bone tissue is not uniform. Studies have shown that Mg^2+^ may be locally enriched in certain functional regions, such as the bone-cartilage interface and areas of newly formed bone [41]. In this study, we designed a series of Mg-BG materials with magnesium doping ratios ranging from 0 to 36%. Although Mg-BG are structurally homogeneous, this gradient design enabled the identification of an optimal doping level that functionally approximates or covers the range of locally elevated Mg^2+^ concentrations potentially present in native bone tissue. Furthermore, the mesoporous structure of Mg-BG was engineered to enable sustained ion release, which is expected to maintain extracellular Mg^2+^ concentrations near physiological levels (0.75–0.95 mmol/L) [22], thereby providing a favorable microenvironment for hDPSCs modulation.

### Porosity and ion release capacity of Mg-BG

**Figure 2:**
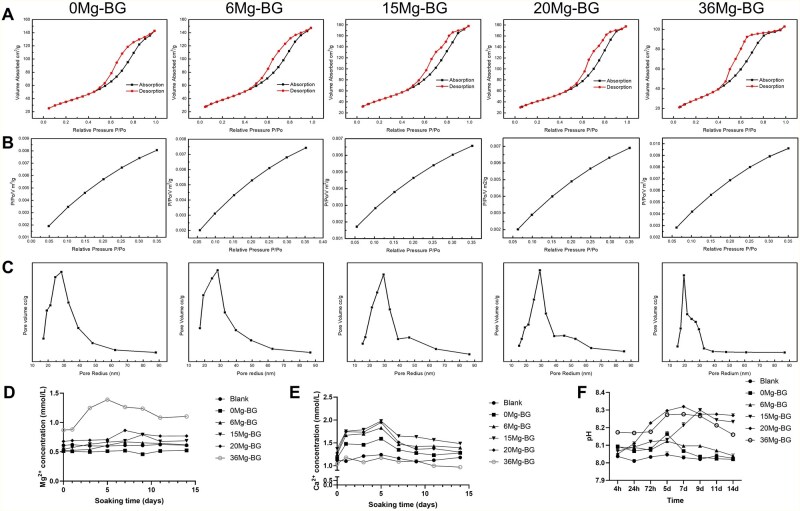
N_2_ Adsorption-desorption isotherms and ion release capacity of Mg-BG powder. (**A**) Nitrogen adsorption-desorption isotherms of Mg-BG powders; (**B**) Relative pressure (*P*/*P*_0_) profile corresponding to the isotherms; (**C**) Pore size distribution curve derived from the adsorption branch; (**D**) Magnesium ion concentration in Mg-BG extract dilutions at various time points; (**E**) Calcium ion concentration in Mg-BG extract dilutions at various time points; (**F**) pH value of Mg-BG extract dilutions measured at different time points.

The surface and pore structure of Mg-BG powders (0 Mg-BG, 6 Mg-BG, 15 Mg-BG, 20 Mg-BG and 36 Mg-BG) were examined using nitrogen adsorption-desorption isotherms. The results demonstrated that all Mg-BG powders exhibited type IV adsorption isotherms and H3-type hysteresis loops (Figure 2A), characteristic of mesoporous materials [42]. The mesopores observed were narrow, formed by the aggregation of glass sol particles, which is consistent with the TEM images. As the magnesium doping concentration increased, the specific surface area, pore volume and pore size of the Mg-BG powders initially increased, then slightly decreased (Figure 2B and C). The 15 Mg-BG and 20 Mg-BG powders exhibited the largest pore size and specific surface area, with specific surface areas of 267.6774 m^2^/g and 255.4998 m^2^/g, and pore radius of 29.20 nm and 29.19 nm, respectively (Supplementary Table S2). The observed variation in pore size and specific surface area of Mg-BG, initially increasing and subsequently decreasing with the increase in magnesium doping, may be due to the smaller ionic radius of magnesium in comparison to calcium. The initial addition of magnesium ions likely disrupts the original calcium-oxygen network, leading to the formation of additional voids or larger pore structures. However, as magnesium progressively replaces calcium, the incorporation of MgO induces changes in the atomic arrangement of the glass network, resulting in a more compact structure [43].

In PBS mixed with standard culture medium at a 1:32 ratio, the release of Mg^2+^, Ca^2+^ and the pH value were monitored continuously for 14 days. The results revealed that the Mg^2+^ concentration in all groups ranged from 0.5 to 1.5 mM. Except for the 36 Mg-BG group, which exhibited significantly higher Mg^2+^ content compared to the other groups, an increase in Mg content resulted in a gradual rise in Mg^2+^ concentration in the solution. During 0–7 days, the Mg^2+^ levels in each group steadily increased, followed by a gradual decline due to crystal formation (Figure 2D). The Ca^2+^ concentration in each solution ranged between 1 and 2 mM, with the Ca^2+^ concentration initially rising and then decreasing as Mg content increased, with the 15 Mg-BG group exhibiting the highest Ca^2+^ concentration. Over time, from 0 to 5 days, Ca^2+^ levels gradually increased in all groups, followed by a slow decline due to crystal formation (Figure 2E). The pH value in all groups remained within a weakly alkaline range, peaking at 5–7 days, before gradually returning to a slightly alkaline state (Figure 2F).

Consistent with the surface area results, we observed that as the amount of Mg added increased, the release of Mg^2+^ from the extract dilution gradually increased. In the range from 0 to 15 Mg-BG, despite the reduction in CaO content, the larger surface area led to a gradual increase in Ca^2+^ release. However, from 15 to 36 Mg-BG, the decrease in CaO content resulted in a gradual decline in Ca^2+^ release. Due to its porous structure, Mg-BG was able to release ions over several days. The pH variation was closely associated with the total concentration of Mg^2+^ and Ca^2+^ in the solution. Between 7 and 14 days, the concentrations of Mg^2+^ and Ca^2+^ decreased slowly, until they reached a stable state. This may be attributed to the supersaturation of the solution, which prompted the deposition of calcium phosphate minerals, such as hydroxyapatite, on the material surface. This deposition reduced the ion release rate [44].

Overall, the ion release from Mg-BG in all groups remained at low concentrations, exhibiting a trend of gradual and continuous release followed by stabilization over the 14-day measurement period. This behavior may be beneficial for achieving concentrations that promote cellular function. However, further experiments are required to identify the specific optimal group.

### Identification of hDPSCs and hDPSCs replicative senescence cell model

To identify hDPSCs, we assessed their adhesion growth, surface markers and osteogenic differentiation potential. The morphology of hDPSCs revealing a fibroblast-like, spindle-shaped appearance with adherence to the plastic surface of the culture dish (Supplementary Figure S1A). Flow cytometry analysis confirmed the presence of MSCs markers, including CD90, CD105, CD106 and CD146, while showing negative expression of hematopoietic markers CD45 and CD14 (Supplementary Figure S1B). The osteogenic differentiation potential of hDPSCs was confirmed through *in vitro* induction assays. After 14 days of osteogenic induction, ARS staining showed the formation of mineralized nodules (Supplementary Figure S1C). These results confirm the successful isolation and cultivation of hDPSCs for further experimentation.

To screen the cell passage number that effectively represents the replicative senescence of hDPSCs and establish a reliable replicative senescence model, we subcultured Passage 4 (P4), Passage 6 (P6), Passage 8 (P8) and Passage 10 (P10) hDPSCs. Inverted microscope observation revealed morphological changes from P4 to P10, which they became flatter, larger and transitioned from a short spindle shape to a longer, more elongated form with slender pseudopodia extending (Supplementary Figure S2A). SA-β-Gal staining showed an increasing proportion of blue-stained senescent cells with each passage, significantly higher in the P8 and P10 groups (Supplementary Figure S2B). qRT-PCR results showed that with increasing passage number, the expression of senescence-related markers *p16*, *p21*, *p53* and SASP components, including *PAI-1* and *IL-6* mRNA, increased effectively, with *IL-8* mRNA also showing an upward trend. Notably, the expression of senescence-related genes increased significantly in the P10 group (Supplementary Figure S2C). These findings indicate that with continued subculture, hDPSCs exhibited clear signs of senescence, consistent with previous studies [45, 46]. Based on these indicators, the P10 group exhibited distinct senescence compared to the P4 group, and the P4 group were selected to represent the young group hDPSCs, while the P10 group represent the replicative senescence group hDPSCs for subsequent experiments.

To verify the relationship between replicative senescence of hDPSCs and osteogenic differentiation capacity, we performed osteogenic differentiation induction culture on the P4, P6, P8 and P10 groups. qRT-PCR results revealed that as the number of generations increased, the mRNA expression levels of osteogenic differentiation markers *ALP*, *Col1*, *OCN*, *RUNX2* and odontogenic differentiation markers *DMP1* and *DSPP* decreased, with osteogenic differentiation marker *OPN* showing an downward trend (Supplementary Figure S3A). ALP staining results demonstrated a significant decrease in the intensity of blue staining with the increase in generations (Supplementary Figure S3B), while ARS staining results showed a marked reduction in the formation of mineralized nodules with the increase in generations (Supplementary Figure S3C). These findings indicate a close relationship between replicative senescence and osteogenic differentiation potential. As the passage number increased, the osteogenic differentiation ability of hDPSCs significantly declined, which is consistent with other studies [47].

### Effects of Mg-BG on biocompatibility and replicative senescence of hDPSCs

Having demonstrated that the ion release capacity of Mg-BG is within the range that could potentially promote cell viability and confirmed that the osteogenic differentiation ability of replicatively senescent hDPSCs is significantly impaired, we aimed to explore the hypothesis that Mg-BG can improve the replicative senescence of hDPSCs. We used the diluted Mg-BG extract as conditioned medium to culture replicatively senescent hDPSCs and observed changes in senescence-related indicators.

Research has demonstrated that the dissolution of BG ions and surface chemical structure factors are the primary mechanisms through which bioactive glass regulates gene expression [48]. Consequently, cytological evaluations of bioactive glass are primarily conducted using leachates, which are considered a scientifically valid method for exploring the biological activity of BG [48]. Our previous research revealed that the extract of BG synthesized by the sol-gel method, mixed with complete culture medium at a ratio of 1:32, exhibits excellent biocompatibility with hDPSCs and most effectively promotes their osteogenic differentiation [37]. Since our Mg-BG is developed based on this BG, we chose this mixing ratio for subsequent research. MTA has been extensively studied and proved to promote bone formation effectively [49], and its combination with hDPSCs can significantly enhance osteogenic differentiation while maintaining good biocompatibility [50]. Given these factors, we used MTA as the positive control group.

**Figure 3:**
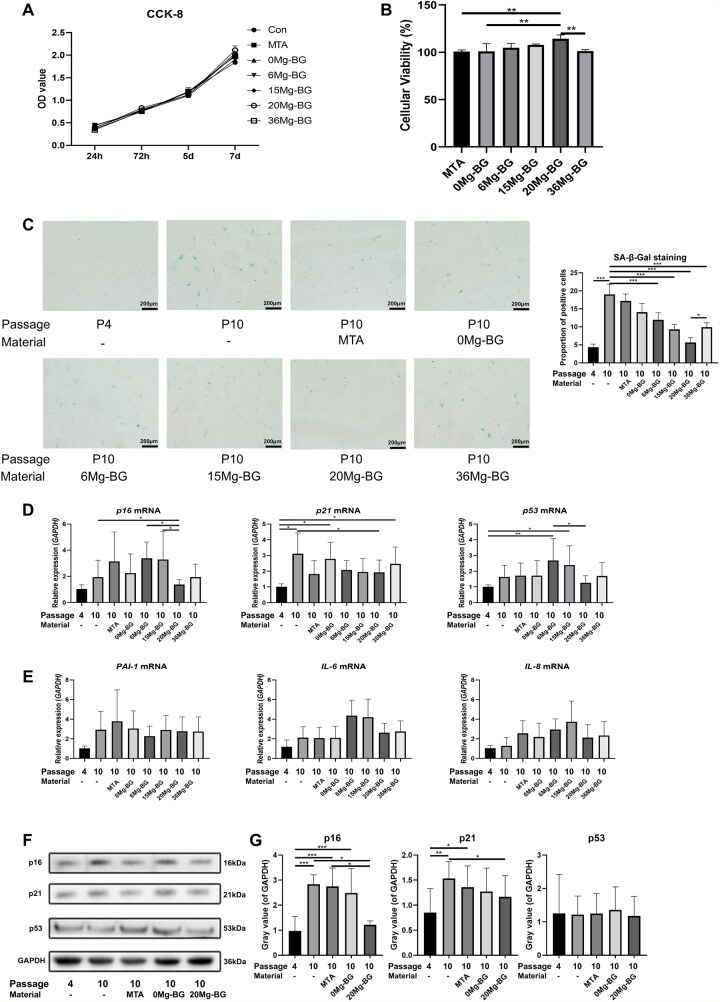
Effects of Mg-BG on replicative senescence of hDPSCs. (**A**) Cell proliferation curves of hDPSCs cultured with Mg-BG for 1, 3, 5 and 7 days; (**B**) Cell viability of hDPSCs cultured with Mg-BG for 3 days; (**C**) Senescence assessment using SA-β-Gal staining, observed by inverted microscope (20× magnification); (**D**) qRT-PCR analysis of senescence-related genes (*p16*, *p21* and *p53*) at the mRNA level; (**E**) qRT-PCR analysis of SASP-related genes (*PAI-1*, *IL-6* and *IL-8*) at the mRNA level; (**F**) Western blot analysis of senescence-associated proteins (p16, p21 and p53); (**G**) Quantitative analysis of protein expression levels for p16, p21 and p53. (*P < 0.05, **P < 0.01 and ***P < 0.001 indicate significant differences between the indicated columns).

The CCK-8 assay was employed to evaluate the cytotoxicity of Mg-BG and MTA extract diluents on hDPSCs. The cell proliferation curves for Mg-BG among all groups and MTA exhibited no significant differences, suggesting that neither Mg-BG nor the MTA extract diluent exerted notable cytotoxic effects on hDPSCs (Figure 3A). Furthermore, the percentage of viable hDPSCs in the 20 Mg-BG group was significantly greater than that of the control group, suggesting that 20 Mg-BG effectively promoted cell proliferation (Figure 3B).

Based on the results from section identification of hDPSCs and hDPSCs replicative senescence cell model, we designated the hDPSCs P4 group as the young group (positive control) and the P10 group as the replicative senescence group (negative control). The other groups consisted of P10 hDPSCs cultured under conditions with Mg-BG or MTA extract diluents, named according to the material (0 Mg-BG, 6 Mg-BG, 15 Mg-BG, 20 Mg-BG, 36 Mg-BG groups). The impact of Mg-BG on the replicative senescence of hDPSCs were evaluated after 7 days of culture. SA-β-Gal staining revealed that, in contrast to the P10 group, the proportion of blue-stained senescent cells in hDPSCs first decreased and then increased with the rising Mg content in Mg-BG. Among the groups, the 20 Mg-BG group exhibited the lowest proportion of blue-stained cells (Figure 3C). qRT-PCR analysis showed that, compared to the P10 group, the expression of senescence-related genes *p16* and *p21* mRNA was significantly reduced in the 20 Mg-BG group, while the expression of *p53* mRNA showed a downward trend (Figure 3D). Furthermore, the expression of the SASP, which refers to the collection of bioactive molecules secreted by senescent cells, was examined by qRT-PCR. The mRNA expression of *PAI-1* demonstrated a downward trend, while *IL-6* and *IL-8* mRNA levels showed no significant differences (Figure 3E). Western blot analysis revealed that in the 20 Mg-BG group, the protein expression of senescence-related genes p16 and p21 was significantly reduced in replicative senescent hDPSCs, while p53 protein expression showed no significant differences across all groups (Figure 3F and G).

Interestingly, our results suggest that Mg-BG alleviates replicative senescence in hDPSCs primarily by downregulating p16 and p21, with minimal involvement of p53. The p16(Ink4a)/Rb pathway and p53/p21 pathway represent two major molecular routes mediating cellular senescence, yet they differ significantly in their activation context and functional outcomes [51]. The p16(Ink4a)/Rb pathway is predominantly associated with replicative senescence triggered by telomere shortening or prolonged proliferation [52], whereas the p53/p21 pathway is more commonly activated by acute cellular stress, such as DNA damage or oxidative insult [53]. Moreover, p21 can also be induced via p53-independent mechanisms, such as the p21(Waf1/Cip1) pathway, to drive cellular senescence in hepatocytes and breast cancer cells [54]. In this study, p53 expression showed no significant change, indicating that Mg-BG has limited impact on stress-induced senescence. The suppression of p16 and p21 likely reflects Mg-BG’s role in relieving cell cycle arrest associated with replicative senescence, suggesting a selective biological effect of Mg^2+^ on senescent hDPSCs.

To further delineate how Mg-BG modulates hDPSCs senescence, we examined not only cell cycle regulators but also SASP-related markers and intracellular pathways. The characteristics of senescent cells vary depending on the specific state of the cell type. Current research identifies several common features of senescent cells, primarily including growth arrest, the expression of antiproliferative molecules such as p16, p21 and p53, and increased lysosomal β-galactosidase activity [55]. However, the SASP, a collection of pro-inflammatory cytokines secreted by senescent cells, may differ between cell types, depending on the distinct activation of damage-sensing signaling pathways, such as the MAPK and NF-κB pathways [56–58]. Our results suggest that Mg-BG may reverse hDPSCs senescence by modulating core characteristics of these cells, including sustained growth arrest, expression of anti-proliferative molecules like p16 and p21, rather than by reducing the secretion of SASP components including IL-6, IL-8 and PAI-1 [58]. Collectively, these findings indicate that 20 Mg-BG effectively promotes hDPSCs proliferation and reduces replicative senescence. However, since Mg-BG does not appear to influence the secretion of SASP factors like IL-6 and IL-8 in hDPSCs, further investigation is necessary to elucidate the behavior of replicatively senescent hDPSCs with respect to the hallmark features of senescence following stimulation with 20 Mg-BG.

### Effects of Mg-BG on the characteristics of replicative senescent hDPSCs

To further explore the effects of Mg-BG on the characteristics of replicative senescence in hDPSCs, we assessed its impact on mitochondrial function, reactive oxygen species (ROS) levels, and characteristic cell surface markers in replicatively senescent hDPSCs. Mitochondria, as the energy centers of cells, are crucial for cellular respiration. However, due to the lack of efficient repair mechanisms, mitochondrial DNA (mtDNA) is more susceptible to mutations than nuclear DNA during the process of cellular proliferation [59]. Mitochondrial dysfunction, a critical hallmark of MSCs senescence, exacerbates senescence through pathways such as the p53/p21 pathway [60]. ROS, metabolic byproducts whose accumulation can accelerate MSCs senescence via various biological processes, including the p21-modulated cell cycle pathway [61], also influence MSC proliferation and differentiation when present in physiological amounts [62]. Additionally, elevated ROS levels contribute to increased mtDNA damage [18]. Surface markers, key criteria for defining MSCs, are specific antigens expressed on the cell surface [63]. However, the expression of these markers can change during cellular senescence. CD90, related to differentiation potential, CD106, associated with adhesion and migration, and CD146, which may be linked to impaired migratory capacity in senescent MSCs, all show a trend of decreased expression with cellular senescence [15, 16]. Therefore, we focused on these functional changes in senescent MSCs and investigated the effects of Mg-BG on these changes in replicatively senescent hDPSCs.

**Figure 4:**
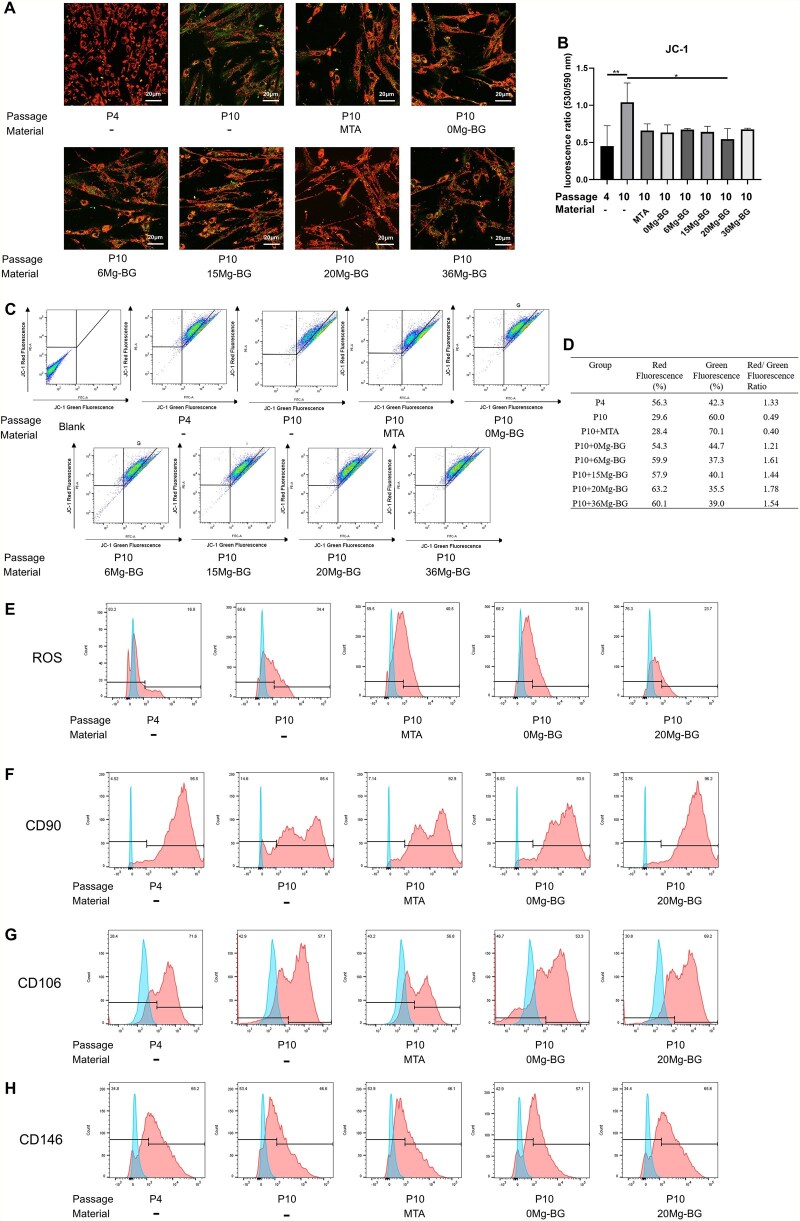
Effects of Mg-BG on mitochondrial function, ROS levels and surface marker expression in replicative senescent hDPSCs. (**A**) JC-1 staining observed by confocal laser scanning microscopy (CLSM) (20× magnification); (**B**) Quantitative analysis of MMP based on CLSM imaging; (**C**) Flow cytometry analysis of JC-1; (**D**) Quantitative analysis of MMP based on flow cytometry results; (**E**) Flow cytometry analysis of ROS levels in hDPSCs; (**F**) Flow cytometry analysis of CD90 expression; (**G**) Flow cytometry analysis of CD106 expression; (**H**) Flow cytometry analysis of CD146 expression. (*P < 0.05, **P < 0.01 and ***P < 0.001 indicate significant differences between the indicated columns).

Mitochondrial membrane potential is a crucial indicator of mitochondrial function [64]. We utilized JC-1 staining to evaluate mitochondrial membrane potential (MMP) levels and employed confocal microscopy and flow cytometry to analyze changes in the MMP of hDPSCs. Compared to the P10 group, the MMP in the Mg-BG group was higher, with the 20 Mg-BG group showing a significant improvement (Figure 4A–D). Based on these results, the 20 Mg-BG group was selected for subsequent experiments. Flow cytometry analysis revealed that ROS levels in the 20 Mg-BG group were significantly lower compared to the P10 group (Figure 4E). Additionally, flow cytometry was utilized to examine the expression of surface markers in hDPSCs across groups. The results indicated that, in comprision to the P10 group, the 20 Mg-BG group effectively enhanced the expression of CD90, CD106 and CD146 (Figure 4F–H).

It is noteworthy that, except for the 36 Mg-BG group, all other Mg-BG formulations significantly improved the MMP of hDPSCs. However, this enhancement did not exhibit a clear dose-dependent trend. This phenomenon may be attributed to the synergistic roles of Mg^2+^ and Ca^2+^ in regulating mitochondrial function: Mg^2+^ helps maintain membrane polarization by inhibiting the opening of the mitochondrial permeability transition pore (mPTP) [65], while Ca^2+^ supports mitochondrial metabolic activity [66]. The restoration of mitochondrial homeostasis in hDPSCs may depend more on the optimal ratio between Mg^2+^ and Ca^2+^ than on increasing Mg^2+^ concentration alone [67]. Moreover, physicochemical characterization showed that 15 Mg-BG and 20 Mg-BG possessed the largest specific surface areas, which may result in more favorable ion release profiles and enhanced cellular uptake of Mg^2+^. In addition, previous studies suggest that MMP changes during cellular senescence follow a nonlinear pattern, MMP declines during early senescence in fibroblasts, temporarily recovers, and then, undergoes depolarization [68]. Therefore, the balanced Mg^2+^/Ca^2+^ ratio and efficient ion release behavior of 20 Mg-BG may position it within an optimal regulatory window, enabling more effective mitochondrial functional restoration and rapid alleviation of senescence in hDPSCs.

Collectively, these findings indicate that the 20 Mg-BG group significantly improved cell functions related to replicative senescence characteristics, including enhanced mitochondrial function, reduced ROS levels and upregulated expression of MSCs key surface markers.

### Effects of Mg-BG on the osteogenic differentiation ability of replicative senescent hDPSCs *in vitro*

Through the experiments conducted in sections Identification of hDPSCs and hDPSCs replicative senescence cell model, Effects of Mg-BG on biocompatibility and replicative senescence of hDPSCs and Effects of Mg-BG on the characteristics of replicative senescent hDPSCs, we have observed that the osteogenic differentiation capacity of replicatively senescent hDPSCs is significantly diminished, whereas 20 Mg-BG can effectively reverse the replicative senescence of hDPSCs and improve their senescence-related characteristics. Consequently, we aim to further investigate whether Mg-BG can effectively restore the osteogenic differentiation capacity of replicatively senescent hDPSCs and identify the optimal group for reversing this decline. For experimental grouping, we replaced the culture medium in all Mg-BG and MTA groups with osteogenic differentiation induction medium. We established standard culture medium groups (P4 group, P10 group) and osteogenic differentiation induction medium groups (P4 Os group, P10 Os group) for P4 and P10, respectively. Additionally, P10 hDPSCs cultured in Mg-BG or MTA extract dilution conditions were named according to the material (0 Mg-BG group, 6 Mg-BG group, 15 Mg-BG group, 20 Mg-BG group, 36 Mg-BG group).

**Figure 5:**
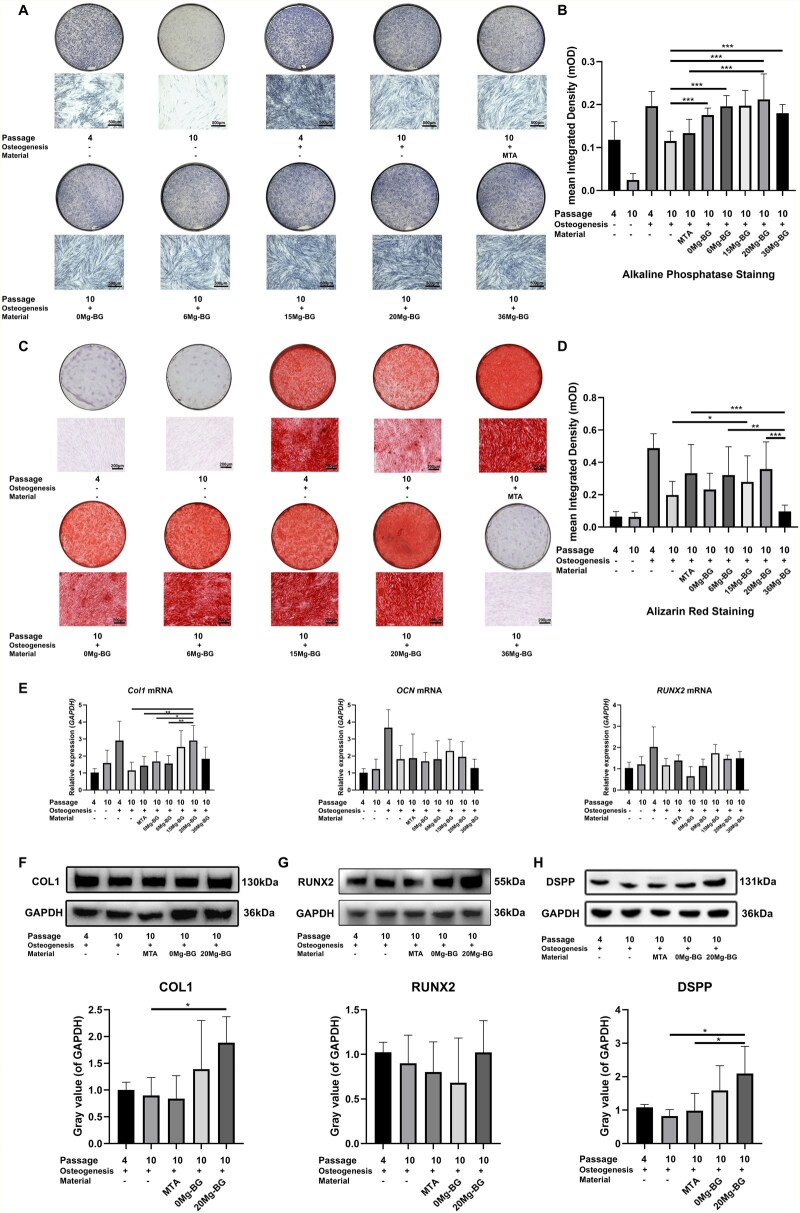
Effects of Mg-BG on osteogenic differentiation in replicative senescent hDPSCs. (**A**) ALP staining after 7 days of osteogenic induction to evaluate early osteogenesis in hDPSCs (10× magnification). (**B**) Semi-quantitative analysis of ALP staining. (**C**) ARS staining after 21 days of osteogenic induction to assess the formation of mineralized nodules (10× magnification). (**D**) Semi-quantitative analysis of ARS staining. (**E**) qRT-PCR analysis of osteogenic differentiation-related genes (*COL1*, *OCN* and *RUNX2*) after 7 days of osteogenic induction. (**F**) Western blot analysis and quantitative analysis of COL1 protein expression after 7 days of osteogenic induction. (**G**) Western blot analysis and quantitative analysis of RUNX2 protein expression after 7 days of osteogenic induction. (**H**) Western blot analysis and quantitative analysis of DSPP protein expression after 7 days of osteogenic induction. (*P < 0.05, **P < 0.01 and ***P < 0.001 indicate significant differences between the indicated columns).

ALP activity is a crucial marker for evaluating early-stage osteogenesis. Therefore, we conducted ALP staining and semiquantitative analysis on hDPSCs from each group after 7 days of culture. Our findings revealed that Mg-BG enhanced ALP expression in replicatively senescent hDPSCs, and the 20 Mg-BG group showing the most notable increase (Figure 5A and B). ARS staining, a key indicator of late-stage osteogenic differentiation, effectively detects mineralized nodule formation. Hence, we assessed the late-stage osteogenic potential of hDPSCs in each group using ARS staining after 21 days of culture, followed by semi-quantitative analysis. The results demonstrated that Mg-BG facilitated mineralized nodule formation in replicatively senescent hDPSCs, with the 20 Mg-BG group exhibiting the most pronounced effect (Figure 5C and D). After 7 days of culture, we used qRT-PCR analysis to examine the expression of osteogenic differentiation-related genes. The qRT-PCR data indicated that, compared to the P10 Os group, the 20 Mg-BG group significantly upregulated *COL1* mRNA expression in replicatively senescent hDPSCs, while the mRNA levels of *OCN* and *RUNX2* showed an increasing trend (Figure 5E). Based on these findings, we selected the P4 Os, P10 Os, MTA, 0 Mg-BG and 20 Mg-BG groups for western blot analysis to assess osteogenic and odontogenic differentiation-related proteins after 7 days of osteogenic differentiation induction. The results revealed that 20 Mg-BG promoted the expression of COL1 and DSPP in replicatively senescent hDPSCs, and the expression of RUNX2 protein in the 20 Mg-BG group showed an increasing trend (Figure 5F–H). In conclusion, the 20 Mg-BG group exhibited superior osteogenic differentiation potential in replicatively senescent hDPSCs. Notably, the reversal of replicative senescence and osteogenic differentiation indicators indicated that the 20 Mg-BG group was the most effective among all groups, emphasizing the pivotal role of reversing replicative senescence in enhancing the osteogenic differentiation capacity of hDPSCs. Based on these results, we selected the 20 Mg-BG group for the subsequent *in vivo* and mechanistic studies.

### Effects of Mg-BG on the osteogenic differentiation ability of replicatively senescent hDPSCs in rats skull defect *in vivo*

To assess the therapeutic potential of Mg-BG in conjunction with replicatively senescent hDPSCs for bone defect treatment, we established a rat skull defect model. Given that biphasic ceramics based on hydroxyapatite/β-tricalcium phosphate (HA/TCP) are widely acknowledged as effective bone substitutes, demonstrating favorable results in numerous clinical studies [69], we selected HA-TCP as a standard negative control scaffold material for both the P4 and P10 groups. Furthermore, as previously noted, both MTA and BG have been reported to promote effective bone defect healing and repair. Consequently, we incorporated HA-TCP, MTA and 0 Mg-BG (BG) as controls to evaluate the impact of Mg-BG on the bone regenerative potential of replicatively senescent hDPSCs. Our experimental groups consisted of P4 hDPSCs+HA-TCP (P4 group), P10 hDPSCs+HA-TCP (P10 group), P10 hDPSCs+MTA (MTA group), P10 hDPSCs + 0Mg-BG (0 Mg-BG group) and P10 hDPSCs + 20Mg-BG (20 Mg-BG group).

A 5-mm diameter defect was created in the rat skull, and bioactive materials, along with either young or replicatively senescent hDPSCs, were transplanted into the bone defect site *in vivo*. Four weeks later, we harvested the heart, liver, spleen, lungs and kidneys to assess the biocompatibility of Mg-BG, and we harvested skulls to assess the defect repair capacity of the transplanted materials combined with hDPSCs in each group.

The H&E staining results of the heart, liver, spleen, lungs and kidneys revealed no significant pathological changes among all groups. All tissues appeared normal, with no evidence of inflammation or tissue damage, indicating good biocompatibility of 20 Mg-BG (Supplementary Figure S4).

**Figure 6:**
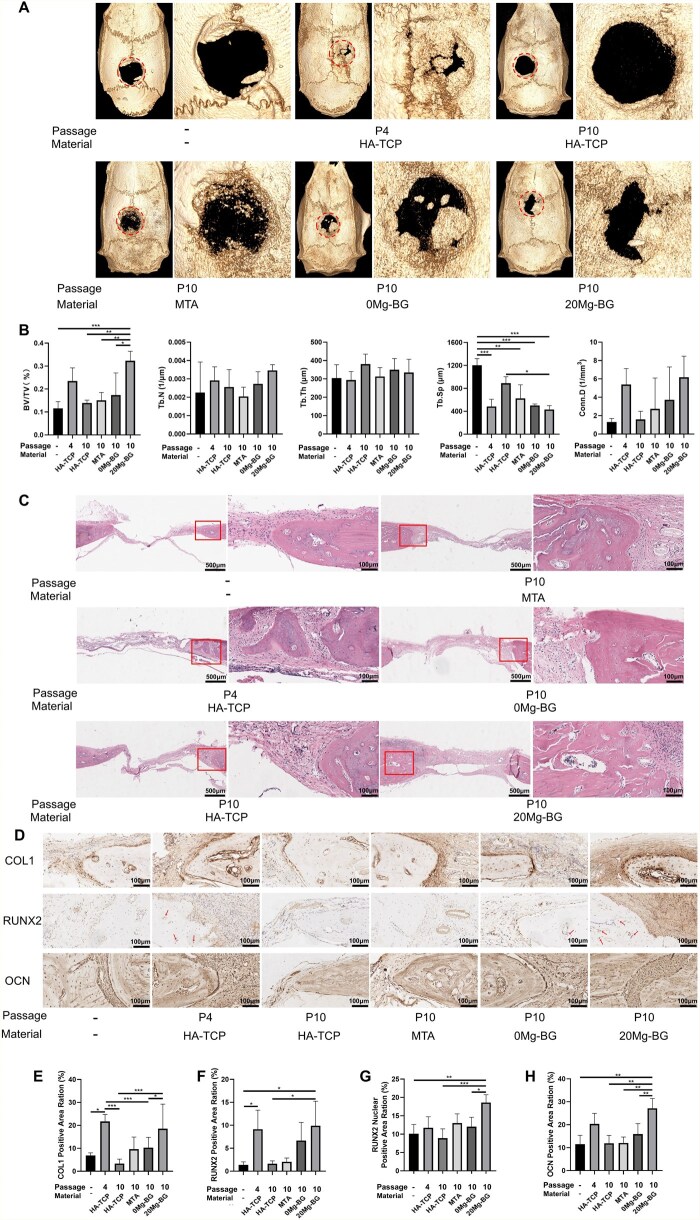
Bone formation analysis at 4 weeks postsurgery. (**A**) Micro-CT images showing the bone formation in the P4 group, P10 group, MTA group, and 0 Mg-BG group, and 20 Mg-BG group; (**B**) Quantitative analysis of BV/TV, Tb.N, Tb.Th, Tb.Sp, and Conn.D based on Micro-CT data; (**C**) H&E staining of the rat cranial defect area showing more extensive newly formed bone tissue in the 20 Mg-BG group (5× and 20× magnification); (**D**) Immunohistochemical images of COL1, RUNX2 and OCN expression in the bone defect area (20× magnification); arrows indicate RUNX2-positive nuclei within cells in bone lacunae; (**E**) Quantification of COL1 expression in the bone defect area; (**F**) Quantification of RUNX2 expression in the bone defect area; (**G**) Quantification of RUNX2 expression based on DAB-positive area fraction; (**H**) Quantification of OCN expression in the bone defect area. (*P < 0.05, **P < 0.01 and ***P < 0.001 indicate significant differences between the indicated columns).

Four weeks post-surgery, micro-CT scanning was performed to evaluate bone formation in each group. The bone volume fraction (BV/TV) indicated that bone anabolism was more pronounced in the 20 Mg-BG group in comparison to the P10, MTA and 0 Mg-BG groups. The trabecular separation (Tb.Sp) in the 20 Mg-BG group was significantly smaller compared to the P10 group. Additionally, the trabecular number (Tb.N) and trabecular thickness (Tb.Th) in the 20 Mg-BG group were generally higher than P10 group, indicating that the reconstructed bone tissue in the 20 Mg-BG group exhibited superior trabecular architecture and mineralization (Figure 6A and B). H&E staining of the rat cranial defect area revealed that the 20 Mg-BG group showed more extensive newly formed bone tissue than P10 group (Figure 6C). Immunohistochemical analysis further demonstrated that the 20 Mg-BG group effectively enhanced the expression of COL1, RUNX2 and OCN in the bone defect area compared to P10 group, providing additional evidence of its superior bone regenerative capacity. RUNX2 staining showed nuclear localization in osteoblast-like cells within bone lacunae, as indicated by arrows in Figure 6D, and the calculated ratio of DAB-positive nuclear area confirmed a significantly higher nuclear localization proportion in the 20 Mg-BG group than in the P10 group (Figure 6D–H).

Notably, the immunohistochemical distribution of COL1, OCN and RUNX2 showed partial deviations from canonical patterns, with overlapping signals between cellular and matrix regions. COL1, normally confined to the extracellular matrix, was predominantly matrix-localized but also faintly detected in some cells [70, 71]. In this study, DAB signals were also observed in certain cellular regions, likely reflecting intracellular procollagen during active synthesis, consistent with previous reports of COL1 presence in early osteoid mineralization or during further mineralization of nascent deposits [72]. During the early stage of mineralization, OCN expected mainly at the mineralization front, exhibited strong interface staining [73]. As mineralization progressed, OCN gradually extended throughout the defect area [74]. In this study, OCN showed strong staining at the mineralization front, with weaker staining observed in other regions. Such a pattern might reflect dynamic mineralization and progressive matrix maturation extending beyond the immediate front, potentially due to early osteoid mineralization or further mineralization, and is consistent with observations in calvarial defects treated with graphene oxide-collagen microgels [75]. RUNX2, classically nuclear, was primarily detected in osteoblast-like cell nuclei within lacunae [76]. However, occasional diffuse matrix staining may be attributed to matrix vesicles or extracellular vesicles that transport RUNX2 or its upstream regulatory molecules, thereby influencing osteogenic signaling within the extracellular matrix [77]. Importantly, quantitative analysis confirmed a significantly higher proportion of nuclear-localized RUNX2 in the 20 Mg-BG group than in P10, underscoring enhanced transcriptional activity. Overall, the IHC staining distribution trends largely conformed to the canonical pattern of mineralization, yet partial deviations were noted, characterized by overlapping signals between cellular and matrix regions. Based on the above analysis, these features likely reflect staged yet temporally overlapping osteogenic processes. In general, this supports the notion that Mg-BG accelerates the coupling of matrix deposition and mineralization coupling.

In summary, the *in vivo* results collectively demonstrate that 20 Mg-BG promotes the repair of bone defects in replicatively senescent hDPSCs. These findings are align with the *in vitro* data, demonstrating that 20 Mg-BG can reverse replicative senescence in hDPSCs and enhance their osteogenic repair ability.

### IKBKGP1-regulated NF-κB pathway may play a crucial role in Mg-BG-mediated reversal of hDPSCs replicative senescence

Based on the above experiments, we have confirmed that 20 Mg-BG can effectively enhance the osteogenic differentiation potential of hDPSCs by reversing their replicative senescence and promote bone defect repair. To investigate the molecular mechanism underlying the reversal of hDPSCs replicative senescence by 20 Mg-BG, we conducted further studies. The experimental groups were as follows: P4 hDPSCs, serving as the positive control group (P4 group), P10 hDPSCs as the negative control group (P10 group) and P10 hDPSCs + 20Mg-BG as the experimental group (20 Mg-BG group). After 7 days of cell culture, mRNA expression was assessed using RNA-Seq technology.

**Figure 7:**
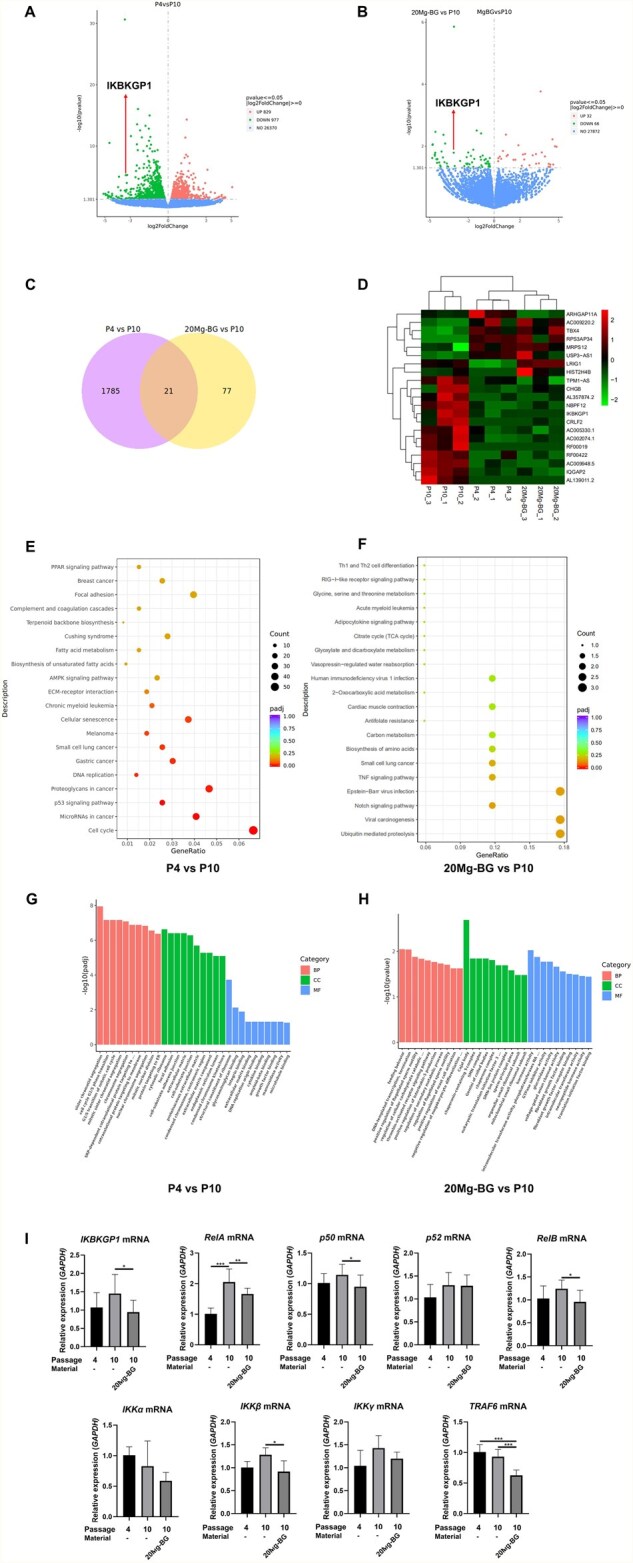
Transcriptome analysis and validation of the mechanism by which 20 Mg-BG reverses replicative senescence of hDPSCs. (**A**) Volcano plot showing DEGs between the P4 and P10 groups; (**B**) Volcano plot showing DEGs between the 20 Mg-BG and P10 groups; (**C**) Venn diagram identifying DEGs between the two comparisons; (**D**) Heat map displaying the expression levels of DEGs across P4, P10 and 20 Mg-BG groups; (**E**) KEGG enrichment analysis of DEGs between the P4 and P10 groups; (**F**) KEGG enrichment analysis of DEGs between the 20 Mg-BG and P10 groups; (**G**) GO enrichment analysis of DEGs between the P4 and P10 groups; (**H**) GO enrichment analysis of DEGs between the 20 Mg-BG and P10 groups; (**I**) qRT-PCR analysis of *IKBKGP1* and NF-κB-related gene expression, including *RelA*, *p50*, *p52, RelB*, *IKKα*, *IKKβ*, *IKKγ* and *TRAF6* mRNA in P4, P10 and 20 Mg-BG groups. (*P < 0.05, **P < 0.01 and ***P < 0.001 indicate significant differences between the indicated columns).

We analyzed the differentially expressed mRNA between the P4 and P10 groups, as well as the differentially expressed mRNA between the 20 Mg-BG and P10 groups. Based on this analysis, we identified the intersection of the two sets of differentially expressed genes (DEGs) for further screening. The volcano plot results indicated that, in comparison to the P10 group, the P4 group had 977 downregulated genes and 829 upregulated genes (P value < 0.05) (Figure 7A). And campared to the P10 group, 20 Mg-BG group exhibited 66 downregulated genes and 32 upregulated genes (Figure 7B). The intersection of these two DEGs sets revealed 21 genes with common differential expression (Figure 7C). Specifically, there were 13 commonly downregulated mRNAs and 8 commonly upregulated mRNAs in the 20 Mg-BG and P4 groups compared to P10 group. The expression levels of these 21 genes across all groups are presented in a heat map (Figure 7D). KEGG pathway analysis indicated that the protein metabolism pathway regulated by ubiquitination was the primary enriched KEGG term in the DEGs between the 20 Mg-BG and P10 groups (Figure 7D and E). GO analysis further suggested that the biological processes of DNA template transcription and termination may play significant roles in this mechanism (Figure 7F and G).

Notably, we observed a significant downregulation of Inhibitor of Nuclear Factor Kappa B Kinase Subunit Gamma Pseudogene 1 (IKBKGP1) expression in both the 20 Mg-BG and P4 group compared to P10 groups. IKBKGP1 is a pseudogene associated with the IKKγ subunit of the IκB kinase (IKK) complex. Consistent with the KEGG analysis, IKBKGP1 function appears to be closely linked to IKKγ and the ubiquitination-regulated Nuclear factor-κB (NF-κB) pathway [78]. As a critical damage-sensing signaling pathway involved in cellular senescence, the NF-κB pathway has been shown to be closely related to cellular senescence [58] and the expression of senescence-related proteins such as p21 and p65 [79]. However, research on IKBKGP1 remains limited.

To verify the role of *IKBKGP1* and the NF-κB pathway in the process of 20 Mg-BG reversing replicative senescence in hDPSCs, qRT-PCR was conducted to assess the expression of *IKBKGP1* and NF-κB-related genes. The qRT-PCR results showed that, in comparison to the P10 group, *IKBKGP1* mRNA expression was significantly reduced in both the 20 Mg-BG and P4 groups. Additionally, NF-κB-related genes such as *RelA*, *p50*, *RelB*, *IKKβ* and *TRAF6* exhibited marked downregulation in the 20 Mg-BG group compared to the P10 group. The mRNA expression of *IKKα* and *IKKγ* also showed a decreasing trend in the 20 Mg-BG group. However, the expression of *p52* mRNA did not differ significantly among the three groups (Figure 7H).

In particular, we noticed that the mRNA expression levels of *IKBKGP1, TRAF6* and *IKKγ* were positively correlated in P10 group and 20 Mg-BG group. IKBKGP1, as a pseudogene related to the IKKγ subunit of the IKK complex, and TRAF6, an E3 ubiquitin ligase that activates IKKγ via its ubiquitination, together suggest that 20 Mg-BG’s modulation of IKBKGP1 may critically influence the ubiquitination and subsequent activation of the IKK complex. Based on this, we speculate that 20 Mg-BG may inhibit the expression of IKBKGP1, further downregulate the expression of the IKKγ complex, and ultimately lead to the inhibition of the NF-κB signaling pathway. However, the specific mode of action between IKBKGP1, TRAF6 and IKKγ is still unclear and needs to be confirmed.

NF-κB is a pivotal transcription factor complex involved in immune and inflammatory responses [80]. Magnesium deficiency induces lipid peroxidation and activates NF-κB in cultured canine cerebrovascular tissue [81]. Furthermore, NF-κB has been implicated in cellular senescence in embryonic fibroblasts [82], with IKKβ contributing to TNF-α-induced proinflammatory activation in MSCs [83]. Additionally, inhibiting NF-κB has been demonstrated to restore osteogenic differentiation in MSCs treated with TNF-α and IL-17 *in vitro* [84], indicating that NF-κB could be a potential therapeutic target for MSCs senescence. Additionally, we observed that 20 Mg-BG notably decreased the expression of RelA and p50, but had no substantial effect on p52. Consequently, we speculate that 20 Mg-BG may inhibit the NF-κB pathway by suppressing the activation of RelA and p50, rather than affecting the RelB pathway.

In summary, the above results suggested that 20 Mg-BG may inhibit the expression of IKBKGP1 in hDPSCs, thereby reducing the ubiquitination activation of IKKα/IKKβ/IKKγ aggregates. Subsequently, by inhibiting the activation of RelA and p50, the NF-κB pathway is suppressed, leading to a reduction in the expression of senescence-associated proteins such as p21, thus, reversing the replicative senescence of hDPSCs. However, further experiments are needed to confirm the effect of 20 Mg-BG on IKKγ ubiquitination after regulating IKBKGP1 expression.

## Discussion

Currently, the clinical application of MSCs shows promising prospects and has been initially recognized by the FDA and other institutions [85]. However, despite significant advances in MSCs-based regenerative medicine, the challenge of achieving a sufficient number of cells remains. Cell senescence, caused by prolonged *in vitro* expansion, continues to seriously hinder the clinical application of MSCs [86]. For decades, researchers have been pursuing strategies to combat “*in vitro* expansion-related senescence” [45]. This is particularly evident in bone defect therapy, where replicative senescence is a major factor diminishing the osteogenic differentiation capacity of *in vitro* expanded MSCs [18].

Traditionally, scaffold materials in bone tissue engineering have been bioinert, designed to be biocompatible with seed cells and the transplanted tissue environment [87]. However, to overcome the limitations of traditional approaches, there is growing emphasis on the bioactivity of scaffold materials, with the goal of enabling them to interact with seed cells and create a synergistic effect where both elements enhance each other’s function [88, 89]. In this context, we propose an innovative strategy: the incorporation of anti-senescence components into the materials used to transplant MSCs into the bone defect area. This approach maximizes the advantage of close contact and continuous interaction between the scaffold and MSCs, simplifying the procedure and allowing the scaffold and MSCs to collaborate in reversing the senescence of MSCs.

In this study, we synthesized Mg-BG with a gradient ratio of Mg doping at the nanoscale and found that, as the Mg doping increased, the crystallization tendency of the powder decreased, resulting in an amorphous structure. All Mg-BG samples exhibited mesoporous structures, with the 15 Mg-BG and 20 Mg-BG powders displaying the largest pore size and specific surface area, facilitating the slow release of Mg^2+^ and Ca^2+^. Notably, the diluted extract of 20 Mg-BG effectively promoted the proliferation of hDPSCs, suggesting that 20 Mg-BG may have a regulatory effect on reversing the replicative senescence of hDPSCs.

Through both *in vitro* and *in vivo* experiments, we demonstrated that Mg-BG can effectively reverse the replicative senescence of hDPSCs, enhance mitochondrial function associated with hDPSCs senescence, reduce ROS levels and increase the expression of cell surface markers related to differentiation, adhesion and migration. Furthermore, Mg-BG significantly enhances the osteogenic differentiation potential of replicatively senescent hDPSCs. The 20 Mg-BG group was identified as the optimal treatment for reversing hDPSC replicative senescence and improving their osteogenic differentiation capacity.

We also identified for the first time that the IKBKGP1-mediated NF-κB pathway may play a crucial role in the reversal of replicative senescence in hDPSCs by 20 Mg-BG. The function of IKBKGP1 may be closely linked to IKKγ ubiquitination-regulated NF-κB signaling. Our study is the first to reveal that the expression of IKBKGP1 in replicatively senescent hDPSCs was significantly elevated in comparison to that in the young group, and 20 Mg-BG effectively reduced IKBKGP1 expression in replicatively senescent hDPSCs to levels comparable to those in the young group. Additionally, we observed that NF-κB-related molecules which may be downstream targets of IKBKGP1, exhibited similar expression patterns to IKBKGP1. Based on these findings, it is indicated that 20 Mg-BG may rejuvenate replicatively senescent hDPSCs by downregulating IKBKGP1 expression, potentially through modulation of the RelA/P50 axis via the reduction of IKKγ ubiquitination within the NF-κB pathway, thereby attenuating the expression of senescence-associated genes such as p21.

The regulation of cellular senescence involves multiple biological mechanisms. In addition to the phenotypic changes and signaling pathways identified in this study, Mg-BG may also influence stem cell fate by reshaping cellular metabolic states. Cellular senescence is often accompanied by metabolic reprogramming, particularly an imbalance between glycolysis and mitochondrial oxidative phosphorylation (OXPHOS), which leads to excessive ROS accumulation and accelerates the development of senescent phenotypes [90, 91]. In our study, Mg-BG significantly reduced ROS levels and improved mitochondrial function in hDPSCs. We, therefore, speculate that the reversal of replicative senescence in hDPSCs by Mg-BG may also involve suppressing excessive glycolysis and enhancing OXPHOS efficiency, thereby restoring metabolic balance and improving the overall metabolic state. Additionally, activation of the NF-κB pathway is known to promote glycolysis, inhibit OXPHOS and disturb ROS metabolism, contributing to metabolic imbalance and functional decline [92, 93]. Mg-BG was found to downregulate key genes in the NF-κB signaling pathway, further supporting its potential role in restoring metabolic homeostasis. Future studies are warranted to verify this hypothesis and expand our understanding of the anti-senescence mechanisms of Mg-BG.

In conclusion, our study presents a novel strategy to reverse the replicative senescence of MSCs and enhance their therapeutic potential for bone defects. Furthermore, it offers valuable insights into the underlying biological mechanisms. Mg-BG may reverse hDPSCs senescence by negatively regulating the IKBKGP1-mediated NF-κB pathway.

## Conclusion

We developed a magnesium-doped bioactive glass (Mg-BG) with varying magnesium doping ratios and a mesoporous structure, capable of releasing Mg^2+^ ions, which promotes hDPSCs proliferation and exhibits excellent compatibility with bone tissue. Our findings demonstrate that 20 Mg-BG effectively reverses the replicative senescence of hDPSCs, enhances the mitochondrial function of replicatively senescent hDPSCs, reduces ROS levels and increases the expression of surface markers associated with differentiation, migration and adhesion. Furthermore, 20 Mg-BG significantly improves both the *in vitro* osteogenic differentiation potential and the *in vivo* cranial repair capacity of replicatively senescent hDPSCs. We also noted a marked increase in the expression of IKBKGP1 in replicatively senescent hDPSCs, which 20 Mg-BG effectively downregulated. The NF-κB signaling pathway, modulated by IKBKGP1, appears to play a key role in the reversal of hDPSCs replicative senescence by 20 Mg-BG. In conclusion, our study provides a new design strategy for anti-senescent biomaterials for bone tissue regeneration.

## Supplementary Material

rbaf105_Supplementary_Data

## References

[1] Sisto JM. Reconstructing jaw defects. J Am Dent Assoc 2017;148:284. 28449741 10.1016/j.adaj.2017.03.009

[2] Zhang S , Zhang X, Li Y, Mao X, Liu R, Qi Y, Lee ES, Jiang HB. Clinical reference strategy for the selection of treatment materials for maxillofacial bone transplantation: a systematic review and network meta-analysis. Tissue Eng Regen Med 2022;19:437–50. 35532735 10.1007/s13770-022-00445-5 PMC9130380

[3] Divieti Pajevic P , Krause DS. Osteocyte regulation of bone and blood. Bone 2019;119:13–8. 29458123 10.1016/j.bone.2018.02.012 PMC6095825

[4] Urban IA , Monje A. Guided bone regeneration in alveolar bone reconstruction. Oral Maxillofac Surg Clin North Am 2019;31:331–8. 30947850 10.1016/j.coms.2019.01.003

[5] Bunpetch V , Zhang ZY, Zhang X, Han S, Zongyou P, Wu H, Hong-Wei O. Strategies for MSC expansion and MSC-based microtissue for bone regeneration. Biomaterials 2019;196:67–79. 29602560 10.1016/j.biomaterials.2017.11.023

[6] Xie C , Liang R, Ye J, Peng Z, Sun H, Zhu Q, Shen X, Hong Y, Wu H, Sun W, Yao X, Li J, Zhang S, Zhang X, Ouyang H. High-efficient engineering of osteo-callus organoids for rapid bone regeneration within one month. Biomaterials 2022;288:121741. 36031458 10.1016/j.biomaterials.2022.121741

[7] Lee YC , Chan YH, Hsieh SC, Lew WZ, Feng SW. Comparing the osteogenic potentials and bone regeneration capacities of bone marrow and dental pulp mesenchymal stem cells in a rabbit calvarial bone defect model. Int J Mol Sci 2019;20:5015. 31658685 10.3390/ijms20205015 PMC6834129

[8] Oliveira NK , Salles THC, Pedroni AC, Miguita L, D'avila MA, Marques MM, Deboni MCZ. Osteogenic potential of human dental pulp stem cells cultured onto poly-epsilon-caprolactone/poly (rotaxane) scaffolds. Dent Mater 2019;35:1740–9. 31543375 10.1016/j.dental.2019.08.109

[9] Zhai Q , Dong Z, Wang W, Li B, Jin Y. Dental stem cell and dental tissue regeneration. Front Med 2019;13:152–9. 29971640 10.1007/s11684-018-0628-x

[10] Wang W , Yan M, Aarabi G, Peters U, Freytag M, Gosau M, Smeets R, Beikler T. Cultivation of cryopreserved human dental pulp stem cells-A new approach to maintaining dental pulp tissue. Int J Mol Sci 2022;23:11485. 36232787 10.3390/ijms231911485 PMC9570360

[11] Namjoynik A , Islam MA, Islam M. Evaluating the efficacy of human dental pulp stem cells and scaffold combination for bone regeneration in animal models: a systematic review and meta-analysis. Stem Cell Res Ther 2023;14:132. 37189187 10.1186/s13287-023-03357-w PMC10186750

[12] Wang L , Fan H, Zhang ZY, Lou AJ, Pei GX, Jiang S, Mu TW, Qin JJ, Chen SY, Jin D. Osteogenesis and angiogenesis of tissue-engineered bone constructed by prevascularized beta-tricalcium phosphate scaffold and mesenchymal stem cells. Biomaterials 2010;31:9452–61. 20869769 10.1016/j.biomaterials.2010.08.036

[13] Yao L , Li F, Yu C, Wang H, Wang Y, Ye L, Yu F. Chronological and replicative aging of CD51(+)/PDGFR-alpha(+) pulp stromal cells. J Dent Res 2023;102:929–37. 36919905 10.1177/00220345231158038

[14] Aslam N , Abusharieh E, Abuarqoub D, Alhattab D, Jafar H, Alshaer W, Masad RJ, Awidi AS. An In vitro comparison of anti-tumoral potential of Wharton’s jelly and bone marrow mesenchymal stem cells exhibited by cell cycle arrest in glioma cells (U87MG). Pathol Oncol Res 2021;27:584710. 34257532 10.3389/pore.2021.584710 PMC8262206

[15] Jung EM , Kwon O, Kwon KS, Cho YS, Rhee SK, Min JK, Oh DB. Evidences for correlation between the reduced VCAM-1 expression and hyaluronan synthesis during cellular senescence of human mesenchymal stem cells. Biochem Biophys Res Commun 2011;404:463–9. 21144825 10.1016/j.bbrc.2010.12.003

[16] Wangler S , Menzel U, Li Z, Ma J, Hoppe S, Benneker LM, Alini M, Grad S, Peroglio M. CD146/MCAM distinguishes stem cell subpopulations with distinct migration and regenerative potential in degenerative intervertebral discs. Osteoarthritis Cartilage 2019;27:1094–105. 31002939 10.1016/j.joca.2019.04.002

[17] Yang YK , Ogando CR, Wang See C, Chang TY, Barabino GA. Changes in phenotype and differentiation potential of human mesenchymal stem cells aging in vitro. Stem Cell Res Ther 2018;9:131. 29751774 10.1186/s13287-018-0876-3 PMC5948736

[18] Weng Z , Wang Y, Ouchi T, Liu H, Qiao X, Wu C, Zhao Z, Li L, Li B. Mesenchymal stem/stromal cell senescence: hallmarks, mechanisms, and combating strategies. Stem Cells Transl Med 2022;11:356–71. 35485439 10.1093/stcltm/szac004 PMC9052415

[19] Morsczeck C. Cellular senescence in dental pulp stem cells. Arch Oral Biol 2019;99:150–5. 30685471 10.1016/j.archoralbio.2019.01.012

[20] Fernandez MA , Achten J, Parsons N, Griffin XL, Png ME, Gould J, Mcgibbon A, Costa ML, Investigators W H. Cemented or uncemented hemiarthroplasty for intracapsular hip fracture. N Engl J Med 2022;386:521–30. 35139272 10.1056/NEJMoa2108337

[21] De Baaij JH , Hoenderop JG, Bindels R. J. Magnesium in man: implications for health and disease. Physiol Rev 2015;95:1–46. 25540137 10.1152/physrev.00012.2014

[22] Barbagallo M , Veronese N, Dominguez LJ. Magnesium in aging, health and diseases. Nutrients 2021;13:463. 33573164 10.3390/nu13020463 PMC7912123

[23] Mccarty MF , Lerner A, Dinicolantonio JJ, Iloki-Assanga SB. High intakes of bioavailable phosphate may promote systemic oxidative stress and vascular calcification by boosting mitochondrial membrane Potential-Is good magnesium status an antidote? Cells 2021;10:1744. 34359914 10.3390/cells10071744 PMC8303439

[24] Veronese N , Pizzol D, Smith L, Dominguez LJ, Barbagallo M. Effect of magnesium supplementation on inflammatory parameters: a meta-analysis of randomized controlled trials. Nutrients 2022;14:679. 35277037 10.3390/nu14030679 PMC8838086

[25] Bussiere FI , Gueux E, Rock E, Girardeau JP, Tridon A, Mazur A, Rayssiguier Y. Increased phagocytosis and production of reactive oxygen species by neutrophils during magnesium deficiency in rats and inhibition by high magnesium concentration. Br J Nutr 2002;87:107–13. 11895162 10.1079/BJN2001498

[26] Zusso M , Lunardi V, Franceschini D, Pagetta A, Lo R, Stifani S, Frigo AC, Giusti P, Moro S. Ciprofloxacin and levofloxacin attenuate microglia inflammatory response via TLR4/NF-kB pathway. J Neuroinflammation 2019;16:148. 31319868 10.1186/s12974-019-1538-9 PMC6637517

[27] Arancibia-Hernandez YL , Hernandez-Cruz EY, Pedraza-Chaverri J. Magnesium (Mg(2+)) deficiency, not well-recognized non-infectious pandemic: origin and consequence of chronic inflammatory and oxidative Stress-Associated diseases. Cell Physiol Biochem 2023;57:1–23. 10.33594/000000603 36722148

[28] Liu M , Dudley SC. Jr. Magnesium, oxidative stress, inflammation, and cardiovascular disease. Antioxidants (Basel) 2020;9:907. 32977544 10.3390/antiox9100907 PMC7598282

[29] Dominguez LJ , Veronese N, Barbagallo M. Magnesium and the hallmarks of aging. Nutrients 2024;16:496. 38398820 10.3390/nu16040496 PMC10892939

[30] Gupta S , Majumdar S, Krishnamurthy S. Bioactive glass: a multifunctional delivery system. J Control Release 2021;335:481–97. 34087250 10.1016/j.jconrel.2021.05.043

[31] Motta C , Cavagnetto D, Amoroso F, Baldi I, Mussano F. Bioactive glass for periodontal regeneration: a systematic review. BMC Oral Health 2023;23:264. 37158885 10.1186/s12903-023-02898-z PMC10169491

[32] El-Rashidy AA , Roether JA, Harhaus L, Kneser U, Boccaccini AR. Regenerating bone with bioactive glass scaffolds: a review of in vivo studies in bone defect models. Acta Biomater 2017;62:1–28. 28844964 10.1016/j.actbio.2017.08.030

[33] Simila HO , Boccaccini AR. Sol-gel bioactive glass containing biomaterials for restorative dentistry: a review. Dent Mater 2022;38:725–47. 35300871 10.1016/j.dental.2022.02.011

[34] Dezfuli SN , Huan Z, Mol A, Leeflang S, Chang J, Zhou J. Advanced bredigite-containing magnesium-matrix composites for biodegradable bone implant applications. Mater Sci Eng C Mater Biol Appl 2017;79:647–60. 28629064 10.1016/j.msec.2017.05.021

[35] Glenske K , Donkiewicz P, Kowitsch A, Milosevic-Oljaca N, Rider P, Rofall S, Franke J, Jung O, Smeets R, Schnettler R, Wenisch S, Barbeck M. Applications of metals for bone regeneration. Int J Mol Sci 2018;19:826. 29534546 10.3390/ijms19030826 PMC5877687

[36] Weng L , Webster TJ. Nanostructured magnesium has fewer detrimental effects on osteoblast function. Int J Nanomedicine 2013;8:1773–81. 23674891 10.2147/IJN.S39031 PMC3652519

[37] Liu C , Zhao F, Zhang W, Chen X. Fabrication and characterization of novel rapid-setting and anti-washout bioactive glass cements for direct pulp capping. Ceram Int 2023;49:17827–37.

[38] Bellucci D , Sola A, Cannillo V. Hydroxyapatite and tricalcium phosphate composites with bioactive glass as second phase: state of the art and current applications. J Biomed Mater Res A 2016;104:1030–56. 26646669 10.1002/jbm.a.35619

[39] Thamma U , Kowal TJ, Falk MM, Jain H. Nanostructure of bioactive glass affects bone cell attachment via protein restructuring upon adsorption. Sci Rep 2021;11:5763. 33707489 10.1038/s41598-021-85050-7 PMC7952393

[40] Shah FA. Revisiting the physical and chemical nature of the mineral component of bone. Acta Biomater 2025;196:1–16. 39892685 10.1016/j.actbio.2025.01.055

[41] Grunewald TA , Rennhofer H, Hesse B, Burghammer M, Stanzl-Tschegg SE, Cotte M, Loffler JF, Weinberg AM, Lichtenegger HC. Magnesium from bioresorbable implants: distribution and impact on the nano- and mineral structure of bone. Biomaterials 2016;76:250–60. 26546917 10.1016/j.biomaterials.2015.10.054

[42] Coleman NJ , Hench LL. A gel-derived mesoporous silica reference material for surface analysis by gas sorption 1. Textural features. Ceram Int 2000;26:171–8.

[43] Barrioni BR , Oliveira AC, De Fátima Leite M, De Magalhães Pereira M. Sol–gel-derived manganese-releasing bioactive glass as a therapeutic approach for bone tissue engineering. J Mater Sci 2017;52:8904–27.

[44] Li J , Li J, Wei Y, Xu N, Li J, Pu X, Wang J, Huang Z, Liao X, Yin G. Ion release behavior of vanadium-doped mesoporous bioactive glass particles and the effect of the released ions on osteogenic differentiation of BMSCs via the FAK/MAPK signaling pathway. J Mater Chem B 2021;9:7848–65. 34586154 10.1039/d1tb01479j

[45] Yu F , Yao L, Li F, Wang C, Ye L. Releasing YAP dysfunction-caused replicative toxicity rejuvenates mesenchymal stem cells. Aging Cell 2023;22:e13913. 37340571 10.1111/acel.13913 PMC10497818

[46] Zhang S , Zhang R, Qiao P, Ma X, Lu R, Wang F, Li C, E L, Liu H. Metformin-induced microRNA-34a-3p downregulation alleviates senescence in human dental pulp stem cells by targeting CAB39 through the AMPK/mTOR signaling pathway. Stem Cells Int 2021;2021:6616240. 33505470 10.1155/2021/6616240 PMC7806386

[47] Alraies A , Alaidaroos NY, Waddington RJ, Moseley R, Sloan AJ. Variation in human dental pulp stem cell ageing profiles reflect contrasting proliferative and regenerative capabilities. BMC Cell Biol 2017;18:12. 28148303 10.1186/s12860-017-0128-x PMC5288874

[48] Jell G , Stevens MM. Gene activation by bioactive glasses. J Mater Sci Mater Med 2006;17:997–1002. 17122910 10.1007/s10856-006-0435-9

[49] Valverde TM , Castro EG, Cardoso MH, Martins-Junior PA, Souza LM, Silva PP, Ladeira LO, Kitten GT. A novel 3D bone-mimetic scaffold composed of collagen/MTA/MWCNT modulates cell migration and osteogenesis. Life Sci 2016;162:115–24. 27523047 10.1016/j.lfs.2016.08.003

[50] Chiu YC , Fang HY, Hsu TT, Lin CY, Shie MY. The characteristics of mineral trioxide aggregate/polycaprolactone 3-dimensional scaffold with osteogenesis properties for tissue regeneration. J Endod 2017;43:923–9. 28389072 10.1016/j.joen.2017.01.009

[51] Lopez-Otin C , Blasco MA, Partridge L, Serrano M, Kroemer G. Hallmarks of aging: an expanding universe. Cell 2023;186:243–78. 36599349 10.1016/j.cell.2022.11.001

[52] Kim HY , Kim HS. Podoplanin depletion in tonsil-derived mesenchymal stem cells induces cellular senescence via regulation of the p16(Ink4a)/Rb pathway. Cell Commun Signal 2024;22:323. 38867259 10.1186/s12964-024-01705-8 PMC11167904

[53] Lee H , Massaro M, Abdelfattah N, Baudo G, Liu H, Yun K, Blanco E. Nuclear respiratory factor-1 (NRF1) induction as a powerful strategy to deter mitochondrial dysfunction and senescence in mesenchymal stem cells. Aging Cell 2025;24:e14446. 39720856 10.1111/acel.14446 PMC11984659

[54] Guo J , Ozaki I, Xia J, Kuwashiro T, Kojima M, Takahashi H, Ashida K, Anzai K, Matsuhashi S. PDCD4 knockdown induces senescence in hepatoma cells by up-regulating the p21 expression. Front Oncol 2018;8:661. 30687637 10.3389/fonc.2018.00661 PMC6334536

[55] Muñoz-Espín D , Serrano M. Cellular senescence: from physiology to pathology. Nat Rev Mol Cell Biol 2014;15:482–96. 24954210 10.1038/nrm3823

[56] Yin K , Patten D, Gough S, De Barros Goncalves S, Chan A, Olan I, Cassidy L, Poblocka M, Zhu H, Lun A, Schuijs M, Young A, Martinez-Jimenez C, Halim TYF, Shetty S, Narita M, Hoare M. Senescence-induced endothelial phenotypes underpin immune-mediated senescence surveillance. Genes Dev 2022;36:533–49. 35618311 10.1101/gad.349585.122 PMC9186388

[57] Sayegh S , Fantecelle CH, Laphanuwat P, Subramanian P, Rustin MHA, Gomes DCO, Akbar AN, Chambers ES. Vitamin D(3) inhibits p38 MAPK and senescence-associated inflammatory mediator secretion by senescent fibroblasts that impacts immune responses during ageing. Aging Cell 2024;23:e14093. 38287646 10.1111/acel.14093 PMC11019144

[58] He S , Sharpless NE. Senescence in health and disease. Cell 2017;169:1000–11. 28575665 10.1016/j.cell.2017.05.015 PMC5643029

[59] Niemann J , Johne C, Schroder S, Koch F, Ibrahim SM, Schultz J, Tiedge M, Baltrusch S. An mtDNA mutation accelerates liver aging by interfering with the ROS response and mitochondrial life cycle. Free Radic Biol Med 2017;102:174–87. 27890640 10.1016/j.freeradbiomed.2016.11.035

[60] Jiang P , Du W, Mancuso A, Wellen KE, Yang X. Reciprocal regulation of p53 and malic enzymes modulates metabolism and senescence. Nature 2013;493:689–93. 23334421 10.1038/nature11776 PMC3561500

[61] Shi Y , Li H, Chu D, Lin W, Wang X, Wu Y, Li K, Wang H, Li D, Xu Z, Gao L, Li B, Chen H. Rescuing nucleus pulposus cells from senescence via dual-functional greigite nanozyme to alleviate intervertebral disc degeneration. Adv Sci (Weinh) 2023;10:e2300988. 37400370 10.1002/advs.202300988 PMC10477883

[62] Refeyton A , Labat V, Mombled M, Vlaski-Lafarge M, Ivanovic Z. Functional single-cell analyses of mesenchymal stromal cell proliferation and differentiation using ALDH-activity and mitochondrial ROS content. Cytotherapy 2024;26:813–24. 38661612 10.1016/j.jcyt.2024.04.003

[63] Yan X , Yan F, Mohammed H a G, Liu O. Maxillofacial-Derived mesenchymal stem cells: characteristics and progress in tissue regeneration. Stem Cells Int 2021;2021:5516521. 34426741 10.1155/2021/5516521 PMC8379387

[64] Yu K , Li F, Ye L, Yu F. Accumulation of DNA G-quadruplex in mitochondrial genome hallmarks mesenchymal senescence. Aging Cell 2024;23:e14265. 38955799 10.1111/acel.14265 PMC11464107

[65] Algieri C , Trombetti F, Pagliarani A, Ventrella V, Nesci S. The mitochondrial F(1)F(O)-ATPase exploits the dithiol redox state to modulate the permeability transition pore. Arch Biochem Biophys 2021;712:109027. 34520732 10.1016/j.abb.2021.109027

[66] D'angelo D , Al Saidi A, Ghirardo G, Reane DV, Gasparini N, Rizzuto R, Raffaello A. Impact of PARL-mediated mitochondrial protease activity on calcium regulation. Biochim Biophys Acta Mol Cell Res 2025;1872:119998. 40484322 10.1016/j.bbamcr.2025.119998

[67] Nesci S , Algieri C, Trombetti F, Ventrella V, Fabbri M, Pagliarani A. Sulfide affects the mitochondrial respiration, the Ca(2+)-activated F(1)F(O)-ATPase activity and the permeability transition pore but does not change the Mg(2+)-activated F(1)F(O)-ATPase activity in swine heart mitochondria. Pharmacol Res 2021;166:105495. 33600941 10.1016/j.phrs.2021.105495

[68] Takenaka Y , Inoue I, Nakano T, Ikeda M, Kakinuma Y. Prolonged disturbance of proteostasis induces cellular senescence via temporal mitochondrial dysfunction and subsequent mitochondrial accumulation in human fibroblasts. FEBS J 2022;289:1650–67. 34689411 10.1111/febs.16249

[69] Lima JR , Soares PBF, Pinotti FE, Marcantonio RaC, Marcantonio-Junior E, De Oliveira G. Comparison of the osseointegration of implants placed in areas grafted with HA/TCP and native bone. Microsc Res Tech 2022;85:2776–83. 35397154 10.1002/jemt.24126

[70] Roberts S , Menage J, Sandell LJ, Evans EH, Richardson JB. Immunohistochemical study of collagen types I and II and procollagen IIA in human cartilage repair tissue following autologous chondrocyte implantation. Knee 2009;16:398–404. 19269183 10.1016/j.knee.2009.02.004 PMC2739934

[71] Chen Y , Yang S, Lovisa S, Ambrose CG, Mcandrews KM, Sugimoto H, Kalluri R. Type-I collagen produced by distinct fibroblast lineages reveals specific function during embryogenesis and osteogenesis imperfecta. Nat Commun 2021;12:7199. 34893625 10.1038/s41467-021-27563-3 PMC8664945

[72] Kadler KE , Hill A, Canty-Laird EG. Collagen fibrillogenesis: fibronectin, integrins, and minor collagens as organizers and nucleators. Curr Opin Cell Biol 2008;20:495–501. 18640274 10.1016/j.ceb.2008.06.008 PMC2577133

[73] Silva Sasso G R D , Silva Florencio, De Pizzol-Junior R, Gil JP, Simoes CD, Sasso-Cerri MJ, Cerri E. P S. Additional insights into the role of osteocalcin in osteoblast differentiation and in the early steps of developing alveolar process of rat molars. J Histochem Cytochem 2023;71:689–708. 37953508 10.1369/00221554231211630 PMC10691409

[74] Li J , Lou S, Bian X. Osteocalcin and GPR158: linking bone and brain function. Front Cell Dev Biol 2025;13:1564751. 40337551 10.3389/fcell.2025.1564751 PMC12055796

[75] Zhou C , Luo C, Liu S, Jiang S, Liu X, Li J, Zhang X, Wu X, Sun J, Wang Z. Pearl-inspired graphene oxide-collagen microgel with multi-layer mineralization through microarray chips for bone defect repair. Mater Today Bio 2022;15:100307. 10.1016/j.mtbio.2022.100307 PMC9189211 35706502

[76] Hu J , Song Y, Zhang Y, Yang P, Chen S, Wu Z, Zhang J. Catalpol enhances osteogenic differentiation of human periodontal stem cells and modulates periodontal tissue remodeling in an orthodontic tooth movement rat model. Drug Des Devel Ther 2024;18:4943–60. 10.2147/DDDT.S482969 PMC11546164 39525045

[77] Lin X , He SQ, Shan SK, Xu F, Wu F, Li FX, Zheng MH, Lei LM, Duan JY, Wu YY, Wu YL, Tang KX, Cui RR, Huang B, Yang JJ, Liao XB, Liu J, Yuan LQ. Endothelial cells derived extracellular vesicles promote diabetic arterial calcification via circ_0008362/miR-1251-5p/Runx2 axial. Cardiovasc Diabetol 2024;23:369. 39420345 10.1186/s12933-024-02440-7 PMC11488141

[78] Chen M , Tan MH, Liu J, Yang YM, Yu JL, He LJ, Huang YZ, Sun YX, Qian YQ, Yan K, Dong MY. An efficient molecular genetic testing strategy for incontinentia pigmenti based on single-tube long fragment read sequencing. NPJ Genom Med 2024;9:32. 38811629 10.1038/s41525-024-00421-z PMC11137062

[79] Wu J , Chen Y, Liao Z, Liu H, Zhang S, Zhong D, Qiu X, Chen T, Su D, Ke X, Wan Y, Zhou T, Su P. Self-amplifying loop of NF-kappaB and periostin initiated by PIEZO1 accelerates mechano-induced senescence of nucleus pulposus cells and intervertebral disc degeneration. Mol Ther 2022;30:3241–56. 35619555 10.1016/j.ymthe.2022.05.021 PMC9552911

[80] Zhang Q , Lenardo MJ, Baltimore D. 30 Years of NF-kappaB: a blossoming of relevance to human pathobiology. Cell 2017;168:37–57. 28086098 10.1016/j.cell.2016.12.012 PMC5268070

[81] Altura BM , Gebrewold A, Zhang A, Altura BT. Low extracellular magnesium ions induce lipid peroxidation and activation of nuclear factor-kappa B in canine cerebral vascular smooth muscle: possible relation to traumatic brain injury and strokes. Neurosci Lett 2003;341:189–92. 12697280 10.1016/s0304-3940(03)00134-4

[82] Chou LY , Ho CT, Hung SC. Paracrine senescence of mesenchymal stromal cells involves inflammatory cytokines and the NF-kappaB pathway. Cells 2022;11:3324. 36291189 10.3390/cells11203324 PMC9600401

[83] Böcker W , Docheva D, Prall WC, Egea V, Pappou E, Roßmann O, Popov C, Mutschler W, Ries C, Schieker M. IKK-2 is required for TNF-alpha-induced invasion and proliferation of human mesenchymal stem cells. J Mol Med 2008;86:1183–92. 18600306 10.1007/s00109-008-0378-3

[84] Chang J , Liu F, Lee M, Wu B, Ting K, Zara JN, Soo C, Al Hezaimi K, Zou W, Chen X, Mooney DJ, Wang CY. NF-kappaB inhibits osteogenic differentiation of mesenchymal stem cells by promoting beta-catenin degradation. Proc Natl Acad Sci USA 2013;110:9469–74. 23690607 10.1073/pnas.1300532110 PMC3677422

[85] The U.S. Food and Drug Administration. FDA approves first mesenchymal stromal cell therapy to treat steroid-refractory acute graft-versus-host disease [EB/OL]. Silver Spring, MD, USA: U.S. Food and Drug Administration; 2024. https://www.fda.gov/news-events/press-announcements/fda-approves-first-mesenchymal-stromal-cell-therapy-treat-steroid-refractory-acute-graft-versus-host

[86] Samsonraj RM , Raghunath M, Nurcombe V, Hui JH, Van Wijnen AJ, Cool SM. Concise review: multifaceted characterization of human mesenchymal stem cells for use in regenerative medicine. Stem Cells Transl Med 2017;6:2173–85. 29076267 10.1002/sctm.17-0129 PMC5702523

[87] Anderson JM , Rodriguez A, Chang DT. Foreign body reaction to biomaterials. Semin Immunol 2008;20:86–100. 18162407 10.1016/j.smim.2007.11.004 PMC2327202

[88] Chen D , Liang Z, Su Z, Huang J, Pi Y, Ouyang Y, Luo T, Guo L. Selenium-Doped mesoporous bioactive glass regulates macrophage metabolism and polarization by scavenging ROS and promotes bone regeneration in vivo. ACS Appl Mater Interfaces 2023;15:34378–96. 37404000 10.1021/acsami.3c03446

[89] Koushik TM , Miller CM, Antunes E. Bone tissue engineering scaffolds: function of multi-material hierarchically structured scaffolds. Adv Healthc Mater 2023;12:e2202766. 36512599 10.1002/adhm.202202766 PMC11468595

[90] Wen J , Yi L, Chen L, Xu J, Zhang Y, Cheng Q, Ping H, Wang H, Shuang F, Chai W, Weng T. Short-term DMOG treatment rejuvenates senescent mesenchymal stem cells by enhancing mitochondrial function and mitophagy through the HIF-1alpha/BNIP3 pathway. Stem Cell Res Ther 2025;16:274. 40457488 10.1186/s13287-025-04422-2 PMC12131571

[91] Casey AM , Ryan DG, Prag HA, Chowdhury SR, Marques E, Turner K, Gruszczyk AV, Yang M, Wolf DM, Miljkovic JL, Valadares J, Chinnery PF, Hartley RC, Frezza C, Prudent J, Murphy MP. Pro-inflammatory macrophages produce mitochondria-derived superoxide by reverse electron transport at complex I that regulates IL-1beta release during NLRP3 inflammasome activation. Nat Metab 2025;7:493–507. 39972217 10.1038/s42255-025-01224-x PMC11946910

[92] Sanchez-Bayuela T , Peral-Rodrigo M, Parra-Izquierdo I, Lopez J, Gomez C, Montero O, Perez-Riesgo E, San Roman JA, Butcher JT, Sanchez Crespo M, Garcia-Rodriguez C. Inflammation via JAK-STAT/HIF-1alpha drives metabolic changes in pentose phosphate pathway and glycolysis that support aortic valve cell calcification. Arterioscler Thromb Vasc Biol 2025;45:232–49. 10.1161/ATVBAHA.124.322375 PMC12197843 40308196

[93] Ma D , Zhang Y, Zhang J, Shi J, Gao S, Long F, Wang X, Pu X, Sun J, Liang S, Cannon RD, Villas-Boas S, Han TL. Outer membrane vesicles derived from probiotic *Escherichia coli* Nissle 1917 promote metabolic remodeling and M1 polarization of RAW264.7 macrophages. Front Immunol 2025;16:1501174. 40510339 10.3389/fimmu.2025.1501174 PMC12159019

