# Supplementary material for: Magnesium-doped bioactive glass enhances bone regeneration by reversing replicative senescence of human dental pulp stem cells in bone defect therapy
Source: Regen Biomater. 2025 Oct 25;13:rbaf105. doi: 10.1093/rb/rbaf105 (PMC12872399; doi:10.1093/rb/rbaf105)
Supplement: rbaf105_Supplementary_Data [file rbaf105_supplementary_data.zip › Supplementary data.docx]

**Table S1. Primers used for qRT-PCR**

| Target gene | Sequence |
| --- | --- |
| GAPDH | Forward: GGAGCGAGATCCCTCCAAAAT  Reverse: GGCTGTTGTCATACTTCTCATGG |
| p16 | Forward: GGGTTTTCGTGGTTCACATCC  Reverse: CTAGACGCTGGCTCCTCAGTA |
| p21 | Forward: GCAGACCAGCATGACAGAT  Reverse: GAGACTAAGGCAGAAGATGTAGAG |
| p53 | Forward: GGCCCACTTCACCGTACTAA  Reverse: GTGGTTTCAAGGCCAGATGT |
| PAI-1 | Forward: CAGCCTGAAACTGTCTGAACA  Reverse: TCATCAGCCACTGGAAAGG |
| IL-6 | Forward: AGCCAGAGTCCTTCAGAGAGA  Reverse: GGATGGTCTTGGTCCTTAGCC |
| IL-8 | Forward: CATCTTCACTGATTCTTGGATACC  Reverse: TGTCTGGACCCCAAGGAA |
| COL1 | Forward: AGAGGAAGGAAAGCGAGGAG  Reverse: GGACCAGCAACACCATCTG |
| ALP | Forward: TACACGGTCCTCCTATACGGAA  Reverse: CTCTCGCTCTCGGTAACATC |
| OPN | Forward: CAGCCAGGACTCCATTGACT  Reverse: ACACTATCACCTCGGCCATC |
| OCN | Forward: CTCACACTCCTCGCCCTATT  Reverse: CGCCTGGGTCTCTTCACTAC |
| RUNX2 | Forward: CCTGAACTCTGCACCAAGTC  Reverse: GAGGTGGCAGTGTCATCATC |
| DMP1 | Forward: CACTCAAGATTCAGGTGGCAG  Reverse: TCTGAGATGCGAGACTTCCTAAA |
| DSPP | Forward: AAAGTGGTGTCCTGGTGCAT  Reverse: CCTGGATGCCATTTGCTGTG |
| IKBKGP1 | Forward: tctgtctgctcgaaccactt  Reverse: ggtctggctgcaaaatggaa |
| p50 | Forward: CTGCAGTTTGATGATGAAGA  Reverse: TAGGCGAGTTATAGCCTCAG |
| p52 | Forward: AGAGGCTTCCGATTTCGATATGG  Reverse: GGATAGGTCTTTCGGCCCTTC |
| RelA | Forward: ATGGGCAAGTCAGCTTCCAAA  Reverse: GCCTCAGAATACTGTTGAGCCT |
| RelB | Forward: CCATTGAGCGGAAGATTCAACT  Reverse: CTGCTGGTCCCGATATGAGG |
| IKKα | Forward: ATGAAGAAGTTGAACCATGCCA  Reverse: CCTCCAGAACAGTATTCCATTGC |
| IKKβ | Forward: CTGGCCTTTGAGTGCATCAC  Reverse: CGCTAACAACAATGTCCACCT |
| IKBKγ | Forward: CGGCAGAGCAACCAGATTCT  Reverse: CCTGGCATTCCTTAGTGGCAG |
| TRAF6 | Forward: TTGCCATGAAAAGATGCAGAGG  Reverse: AGCCTGGGCCAACATTCTC |

**Table S2. Specific surface area and pore radius of Mg-BG powders.**

| Sample Name | Specific surface area (m^2^/g) | Specific pore volume (cm^3^/g) | Pore radius (nm) |
| --- | --- | --- | --- |
| 0Mg-BG | 215.18 | 0.219 | 28.36 |
| 6Mg-BG | 237.27 | 0.223 | 28.25 |
| 15Mg-BG | 267.68 | 0.276 | 29.20 |
| 20Mg-BG | 255.50 | 0.281 | 29.19 |
| 36Mg-BG | 185.79 | 0.170 | 19.48 |


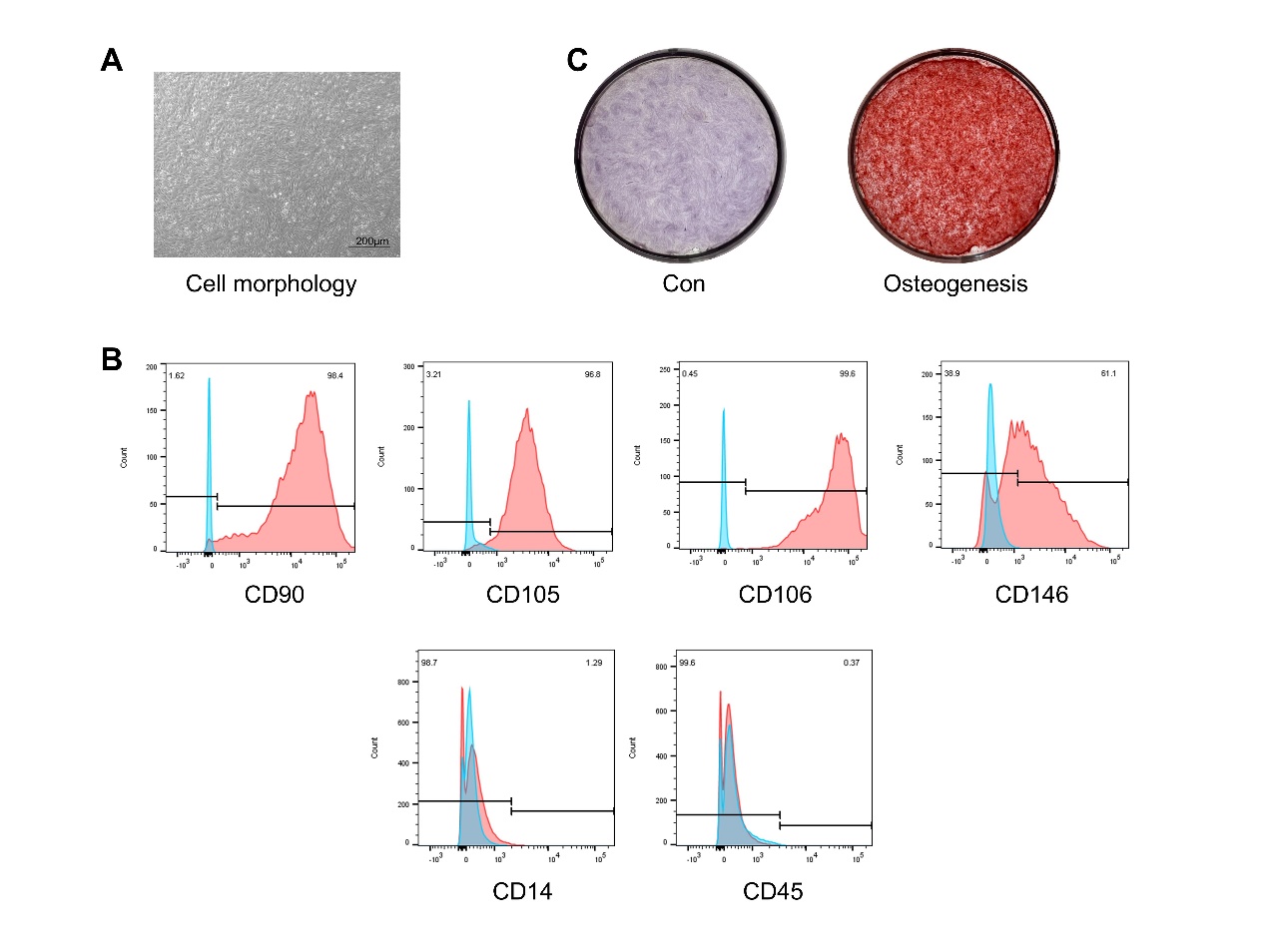


**Figure S1. Characterization of hDPSCs (10× magnification).** (A) Microscopic observation of cell morphology; (B) Low cytometric analysis showing positive expression of CD90, CD105, CD106, and CD146, and negative expression of CD14 and CD45; (C) Alizarin Red S staining for osteogenic differentiation.


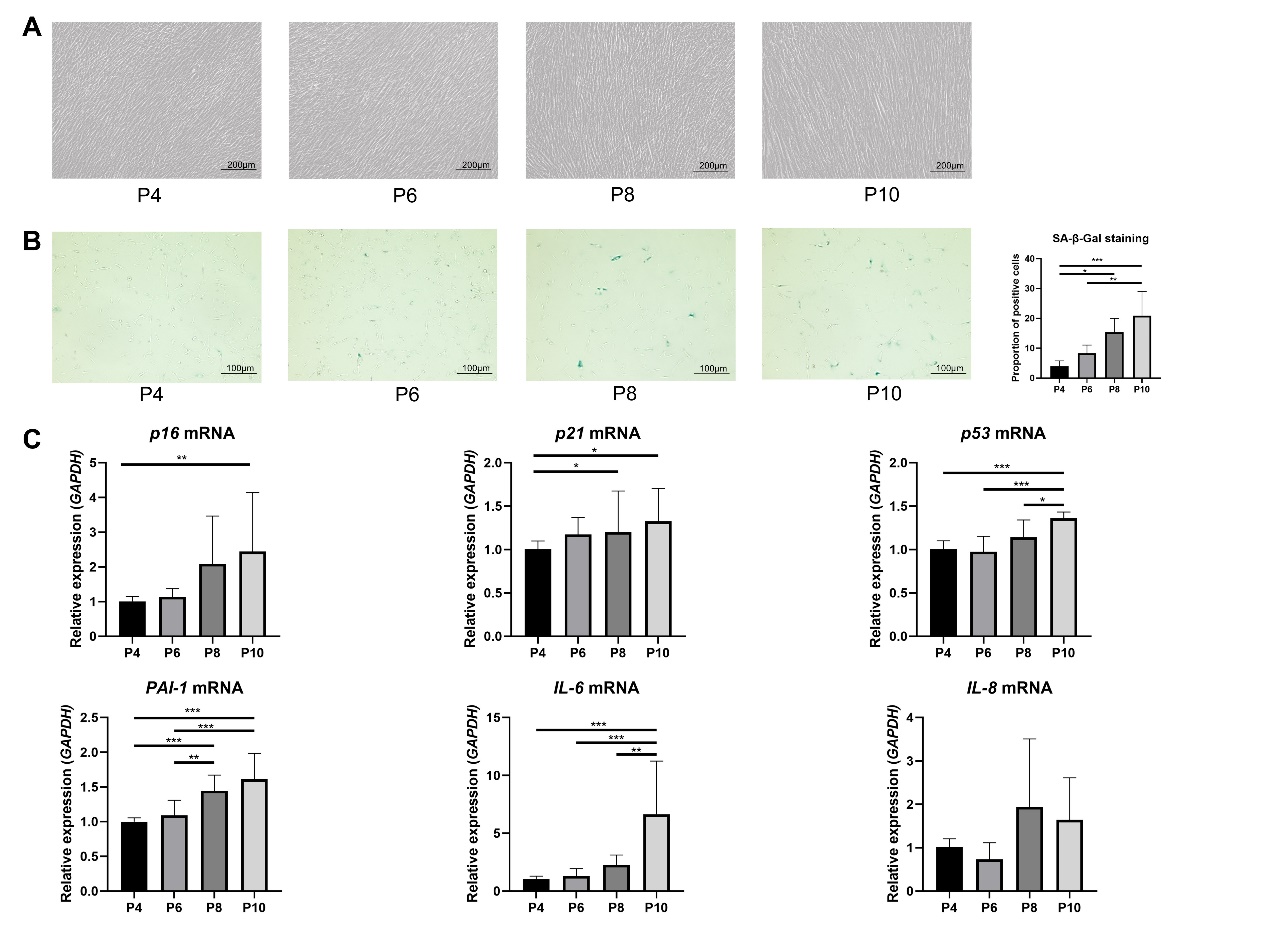
**Figure S2. Identification of the replicative senescence model of hDPSCs.** (A) Morphological changes of hDPSCs at passages P4, P6, P8, and P10 (10× magnification); (B) SA-β-Gal staining to assess hDPSCs cellular senescence at passages P4, P6, P8, and obseved by inverted microscope (20× magnification); (C) qRT-PCR analysis of senescence-associated genes, including *p16, p21, p53, PAI-1*, *IL-6,* and *IL-8* in hDPSCs at passages P4, P6, P8, and P10. (**p* < 0.05, ***p* < 0.01, and ****p* < 0.001 indicate significant differences between the indicated columns).


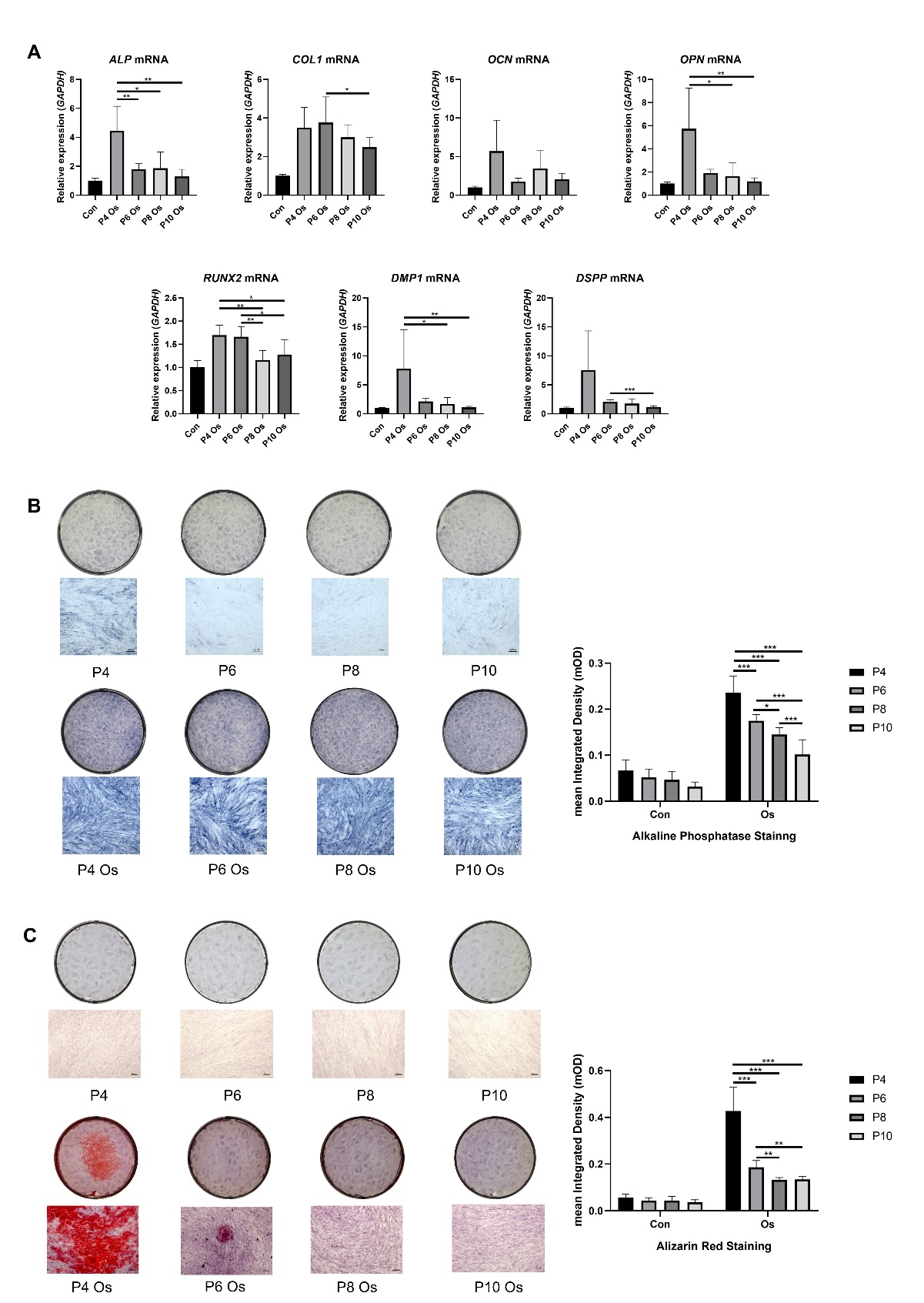


**Figure S3. Assessment of osteogenic differentiation potential in replicative senescent hDPSCs.** (A) qRT-PCR analysis of osteogenic markers (*ALP, COL1, OCN, OPN, RUNX2*) and odontogenic markers (*DMP1, DSPP*) mRNA after 7 days of osteogenic induction in hDPSCs at passages P4, P6, P8, and P10; (B) ALP staining to assess alkaline phosphatase activity after 7 days of osteogenic induction in hDPSCs at passages P4, P6, P8, and P10 (10× magnification); (C) Alizarin Red S (ARS) staining to evaluate mineralization levels after 14 days of osteogenic induction in hDPSCs at passages P4, P6, P8, and P10 (10× magnification). (**p* < 0.05, ***p* < 0.01, and ****p* < 0.001 indicate significant differences between the indicated columns).


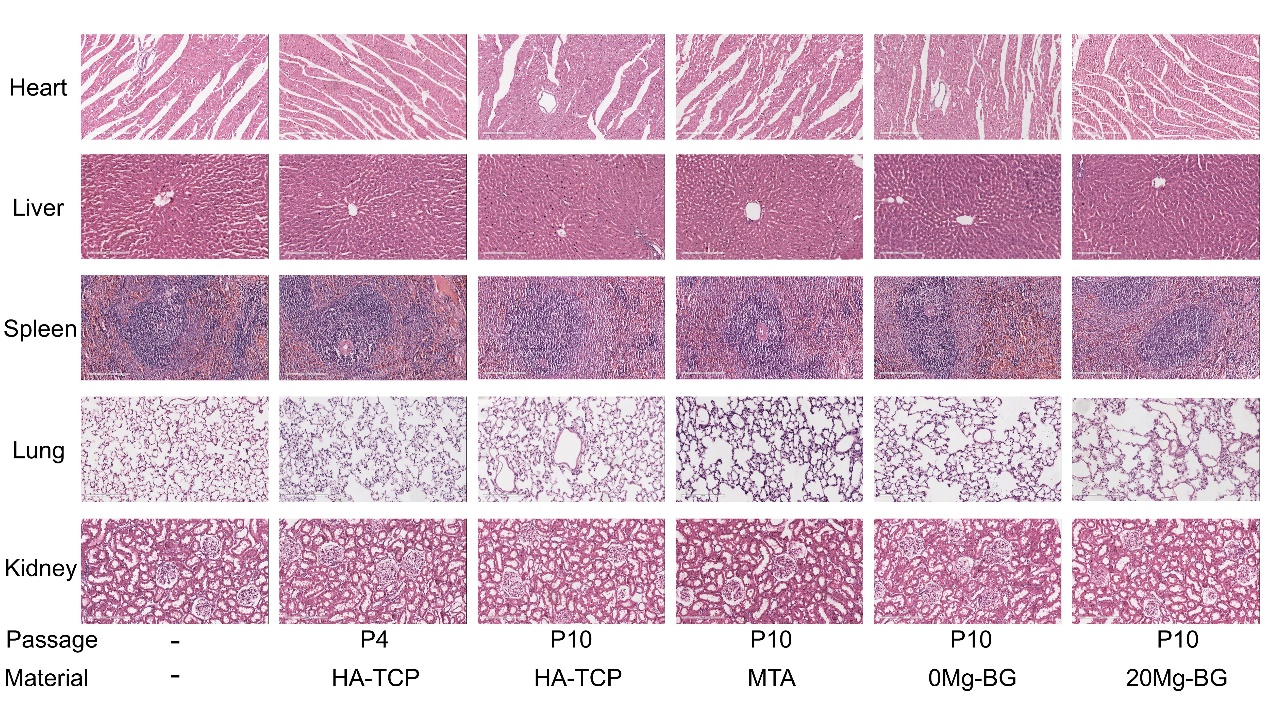


**Figure S4. Histological analysis of tissue biocompatibility. H&E staining of the heart, liver, spleen, lungs, and kidneys (20× magnification).**

**Table S3. Glossary**

| Abbreviation | Definition |
| --- | --- |
| Mg-BG | Magnesium-doped Bioactive Glass |
| BG | Bioactive glass |
| hDPSCs | Human Dental Pulp Stem Cells |
| MSCs | Mesenchymal Stem Cells |
| IL-1 | Interleukin-1 |
| IL-6 | Interleukin-6 |
| IL-8 | Interleukin-8 |
| TNF-α | Tumor Necrosis Factor-α |
| SASP | Senescence-associated Secretory Phenotype |
| ROS | Reactive Oxygen Species |
| TEOS | Tetraethyl Orthosilicate |
| TEP | Triethyl Phosphate |
| CN | Calcium Nitrate |
| MN | Magnesium Nitrate |
| XRD | X-ray Diffraction |
| FTIR | Fourier-transform Infrared Spectroscopy |
| SEM | Scanning Electron Microscopy |
| TEM | Transmission Electron Microscopy |
| ICP-OES | Inductively Coupled Plasma Emission Spectrometer |
| BJH | Barrett-Joyner-Halenda |
| ALP | Alkaline Lhosphatase |
| ARS | Alkaline Red Staining |
| COL1 | Collagen Type I |
| p16 | Cyclin-dependent Kinase Inhibitor p16 |
| p21 | Cyclin-dependent Kinase Inhibitor p21 |
| p53 | Tumor Protein p53 |
| PAI-1 | Plasminogen Activator Inhibitor-1 |
| OCN | Osteocalcin |
| OPN | Osteopontin |
| RUNX2 | Runt-related Transcription Factor 2 |
| DMP1 | Dentin Matrix Acidic Phosphoprotein 1 |
| DSPP | Dentin Sialophosphoprotein |
| IKBKGP1 | Inhibitor of Nuclear Factor Kappa-B Kinase Subunit Gamma Pseudogene 1 |
| p50 | NFκB p50 Subunit |
| p52 | NFκB p52 Subunit |
| RelA | RELA Proto-oncogene |
| RelB | RELB Proto-oncogene |
| IKKα | IκB Kinase α |
| IKKβ | IκB Kinase β |
| IKKγ | IκB Kinase γ |
| TRAF6 | TNF Receptor-Associated Factor 6 |
