# Supplementary figures and images for: Magnesium-doped bioactive glass enhances bone regeneration by reversing replicative senescence of human dental pulp stem cells in bone defect therapy
Source: Regen Biomater. 2025 Oct 25;13:rbaf105. doi: 10.1093/rb/rbaf105 (PMC12872399; doi:10.1093/rb/rbaf105)

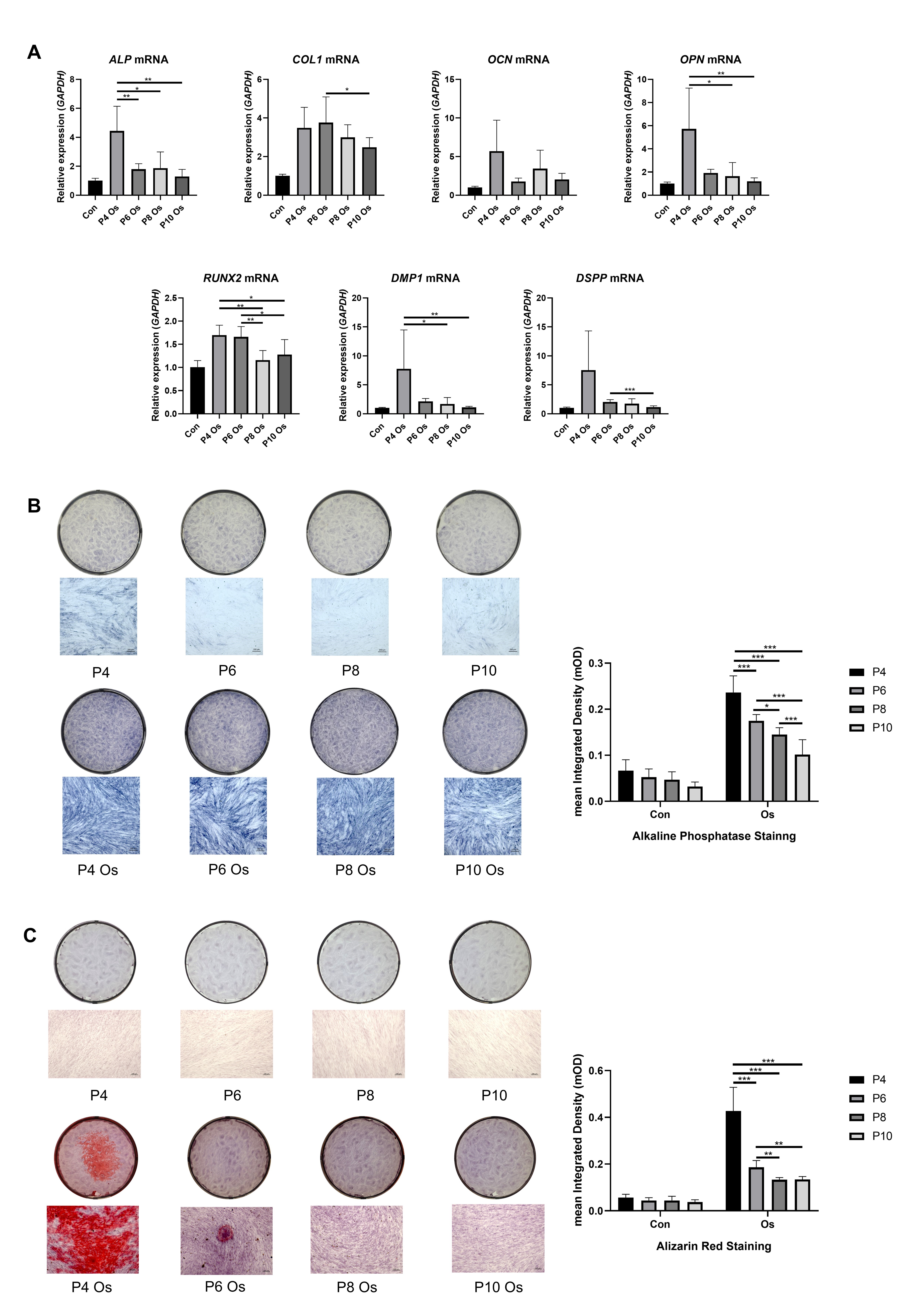

Supplement: rbaf105_Supplementary_Data [file rbaf105_supplementary_data.zip › Figure S3.jpg]

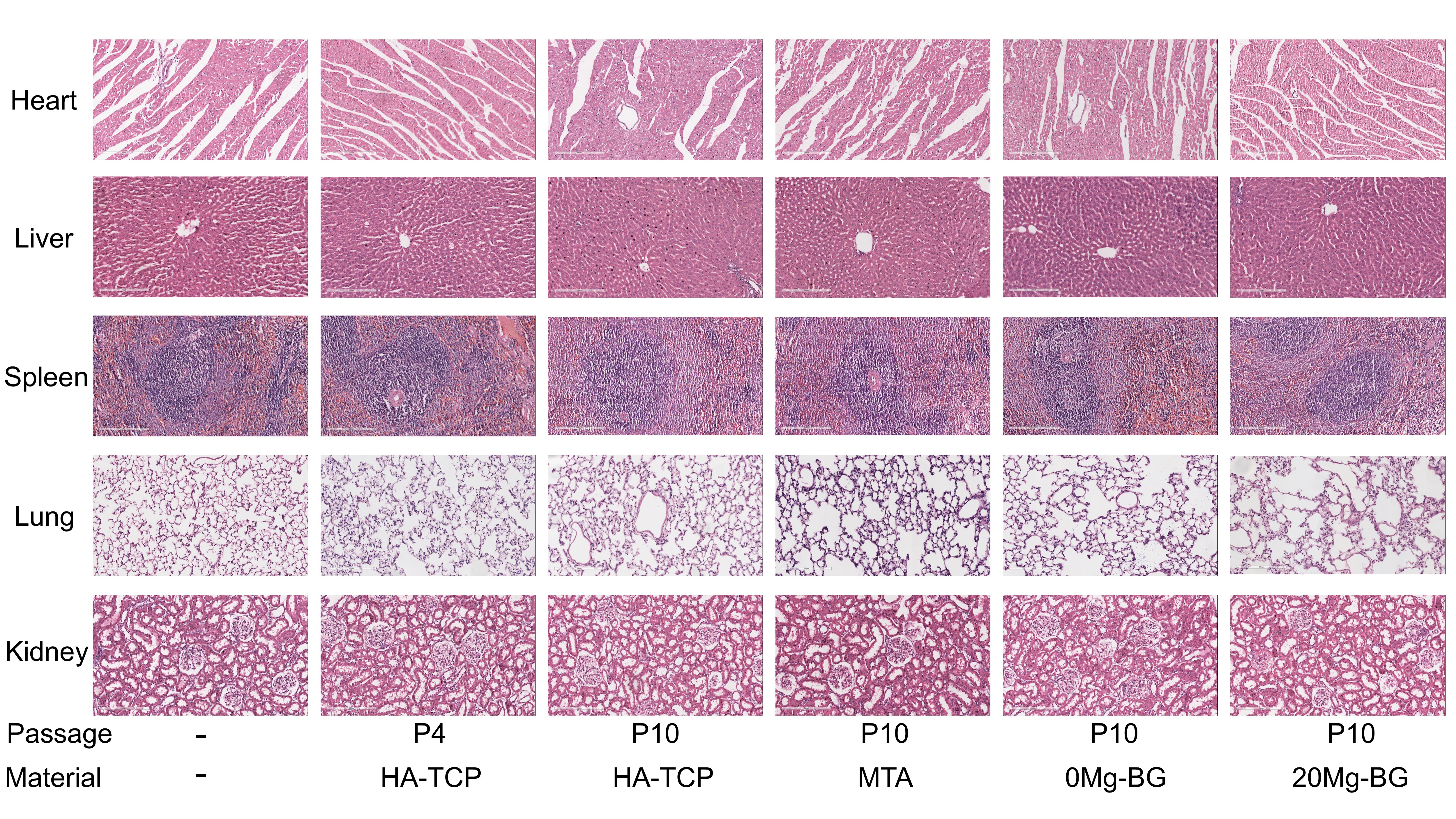

Supplement: rbaf105_Supplementary_Data [file rbaf105_supplementary_data.zip › Figure S4.jpg]

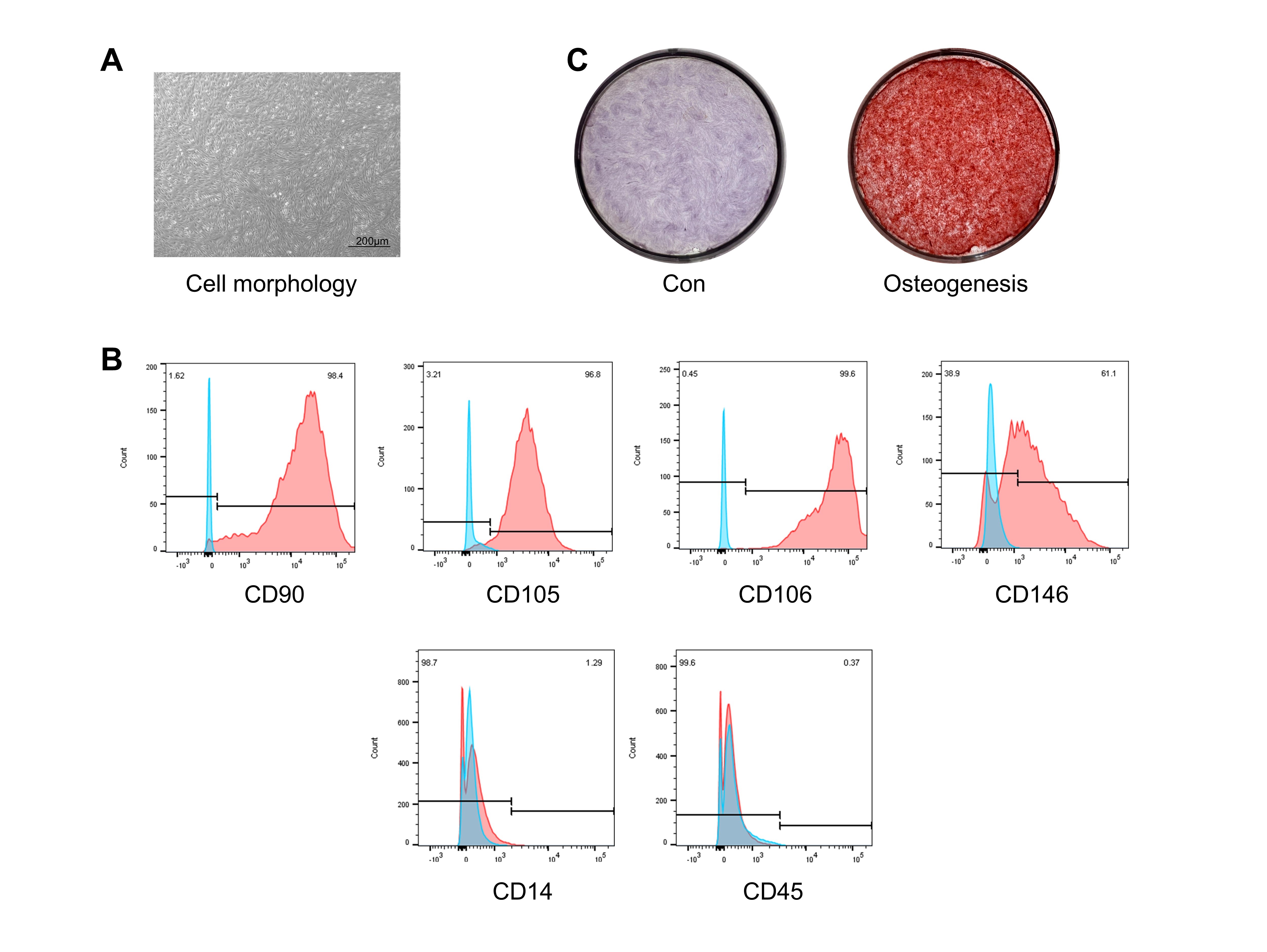

Supplement: rbaf105_Supplementary_Data [file rbaf105_supplementary_data.zip › Figure S1.jpg]

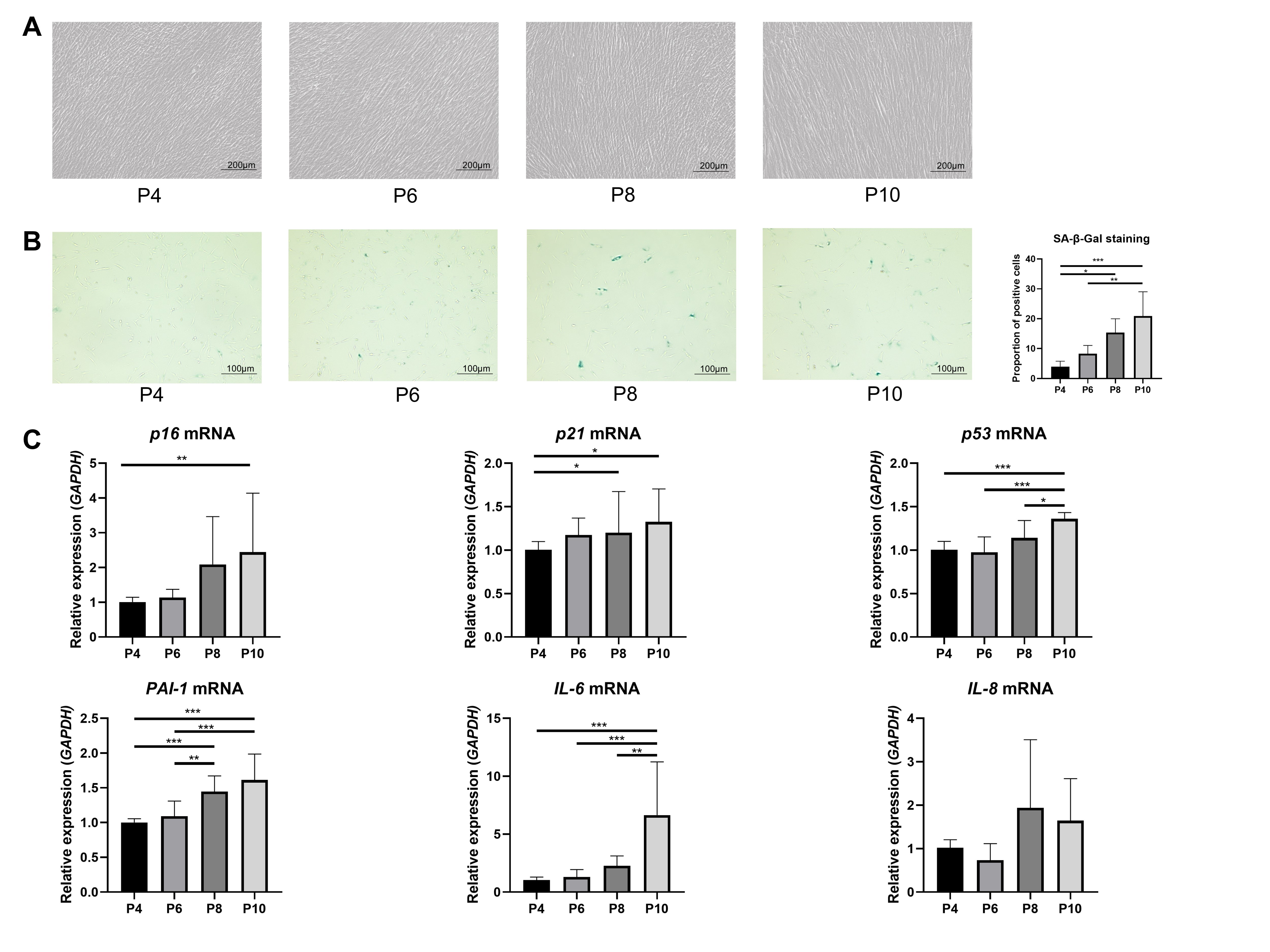

Supplement: rbaf105_Supplementary_Data [file rbaf105_supplementary_data.zip › Figure S2.jpg]
